# Immunoinformatics Approach for Epitope-Based Peptide Vaccine Design and Active Site Prediction against Polyprotein of Emerging Oropouche Virus

**DOI:** 10.1155/2018/6718083

**Published:** 2018-10-08

**Authors:** Utpal Kumar Adhikari, Mourad Tayebi, M. Mizanur Rahman

**Affiliations:** ^1^Department of Biotechnology and Genetic Engineering, Islamic University, Kushtia 7003, Bangladesh; ^2^School of Medicine, Western Sydney University, Campbelltown, NSW, Australia

## Abstract

Oropouche virus (OROV) is an emerging pathogen which causes Oropouche fever and meningitis in humans. Several outbreaks of OROV in South America, especially in Brazil, have changed its status as an emerging disease, but no vaccine or specific drug target is available yet. Our approach was to identify the epitope-based vaccine candidates as well as the ligand-binding pockets through the use of immunoinformatics. In this report, we identified both T-cell and B-cell epitopes of the most antigenic OROV polyprotein with the potential to induce both humoral and cell-mediated immunity. Eighteen highly antigenic and immunogenic CD8^+^ T-cell epitopes were identified, including three 100% conserved epitopes (*TSSWGCEEY*, *CSMCGLIHY*, and *LAIDTGCLY*) as the potential vaccine candidates. The selected epitopes showed 95.77% coverage for the mixed Brazilian population. The docking simulation ensured the binding interaction with high affinity. A total of five highly conserved and nontoxic linear B-cell epitopes “*NQKIDLSQL*,” “*HPLSTSQIGDRC*,” “*SHCNLEFTAITADKIMSL*,” “*PEKIPAKEGWLTFSKEHTSSW*,” and “*HHYKPTKNLPHVVPRYH*” were selected as potential vaccine candidates. The predicted eight conformational B-cell epitopes represent the accessibility for the entered virus. In the posttherapeutic strategy, ten ligand-binding pockets were identified for effective inhibitor design against emerging OROV infection. Collectively, this research provides novel candidates for epitope-based peptide vaccine design against OROV.

## 1. Introduction

Oropouche virus (OROV) is the most common Orthobunyavirus of the Bunyaviridae family, an important causative agent of Oropouche fever in human widespread in South America, especially in Brazil. This fever is clinically characterized as an acute febrile urban arboviral disease [1, 2]. Oropouche fever causes a dengue-like illness with sudden onset of high fever (upwards of 105°F/41°C), headache, malaise, myalgia, arthralgia, nausea, vomiting, and dizziness, allied with viremia in the early five to six days. Usually, the patients recover within two to three weeks without any apparent sequel or death [3]. In some patients, OROV can cause viral meningitis with skin rash, neck stiffness, and photophobia [4]. The OROV can enter into the central nervous system (CNS) and can spread into the brain parenchyma tissue through some infective cycles. Lastly, it causes inflammation and severe manifestations of encephalitis [5]. In spite of its clinical relevance to public health, very little is known about the pathogenesis of OROV in humans and in other vertebrate animals. Proenca-Modena and coworkers demonstrated that the MAVS (mitochondrial antiviral-signaling protein), IRF-3 (interferon regulatory transcription factor) and IRF-7, and MAVS-dependent type I IFN signaling pathway (IFNAR) have dominant roles in restricting OROV infection, and this signaling in nonmyeloid cells greatly contributes to the host defense against orthobunya viruses [6].

OROV was first isolated from the blood of a febrile forest worker in Trinidad & Tobago in 1955. The second strain of OROV was isolated and collected from the pool of 177 *Coquillettidia venezuelensis* mosquitoes in Trinidad in 1960 [1, 7, 8]. Several outbreaks have been recorded in the South Americas, from both urban and rural people of Brazil, Panama, Peru, Ecuador, and Trinidad & Tobago [2]. The virus is deliberated as a public health threat in tropical and subtropical areas of Central and South America, with thirty outbreaks and over half a million (500,000) infected people as of 2005. In 2009, OROV reemerged in the municipalities of Altamira and Santa Barbara, Pará State, and subsequently in Mazagão in the state of Amapá, Brazil [1].

The virus is a single-stranded negative-sense and three-segmented RNA virus. These three segments are large (L), medium (M), and small (S), which encodes the viral RNA-dependent RNA polymerase, the surface glycoproteins (Gn and Gc), and the nucleocapsid (N) protein, respectively [8, 9]. Initially, the partial genome was sequenced for the Brazilian prototype OROV strain BeAn19991, and the complete and corrected genome sequences of the three segments, especially the M segment, were published in 2015 [10, 11]. The M segment comprises a single ORF which encodes a large polyprotein that is cleaved during or after the translation and produces a nonstructural protein (NSm), and two structural surface glycoproteins (Gn and Gc) [2]. These glycoproteins Gn and Gc help in the interaction between the virus particle and the cell receptor. According to Tilston-Lunel et al., the nonstructural protein (NSm) of the M segment is unessential for virus replication in the mosquito and mammalian cell lines [10]. Based on the S segment genetic information, the OROV can consist of four major genotypes (I, II, III, and IV), and Brazil is the main geographical region of these four genotypes [2]. The transmission of OROV is maintained in nature by two distinct cycles such as sylvatic and urban [12]. *Bradypus tridactylus* (sloth), some wild birds, and nonhuman primates (NHPs) control the sylvatic cycle, whereas the urban cycle is maintained only by humans [3, 8]. In humans, the OROV is transmitted by the midge *(Culicoides paraensis)* and the mosquito *(Culex p. quinquefasciatus)*, but no evidence for direct transmission from human to human has ever been recorded [12, 13]. Diagnosis of OROV infection is performed by using various types of classic methods such as virus isolation and cell culture and molecular techniques such as serologic assays (CF, HI, and NT tests and in-house enzyme-linked immunosorbent assay) and real-time polymerase chain reaction (RT-PCR) in acute samples for genome detection [14–16].

Before 2016, there were no reports of the occurrence of Oropouche fever in humans in Goiânia city, the State of Goiás, and central Brazil. But the OROV antibody has been found in NHPs in Goiânia city [17]. So, immunity development in NHPs against this OROV should be taken into consideration as a vital warning sign of ongoing virus circulation.

Since effective and specific commercial rapid diagnostic tests are unavailable, there are great concerns for its possible future outbreaks similar to Zika in South American countries, especially in Brazil. Furthermore, there is no specific approved drug or vaccine against OROV available in the market. Although the role of the MAVS-dependent type I IFN signaling pathway has been shown to control OROV replication [6], it is not enough to completely eradicate OROV from the system. In this situation, adaptive immunity development against OROV could play an effective role in the complete elimination of the virus. Adaptive immunity works based on the recognition of specific epitopes by T-cells after reinfection. Consequently, humoral immune response is activated in humans.

In the present study, we have analyzed the complete proteome of the OROV for the identification of the most antigenic protein and its highest immunogenic and antigenic T-cell epitopes along with B-cell epitopes for the development of the epitope-based peptide vaccine using immunoinformatic approaches. We have also predicted the 3D structure of the most antigenic polyprotein as well as inhibitor-binding sites for the docking simulation study using various bioinformatics tools. Ultimately, this study is aimed at assisting the future laboratory efforts in developing effective vaccination for the prevention of OROV infection.

## 2. Materials and Methods

**Figure 1:**
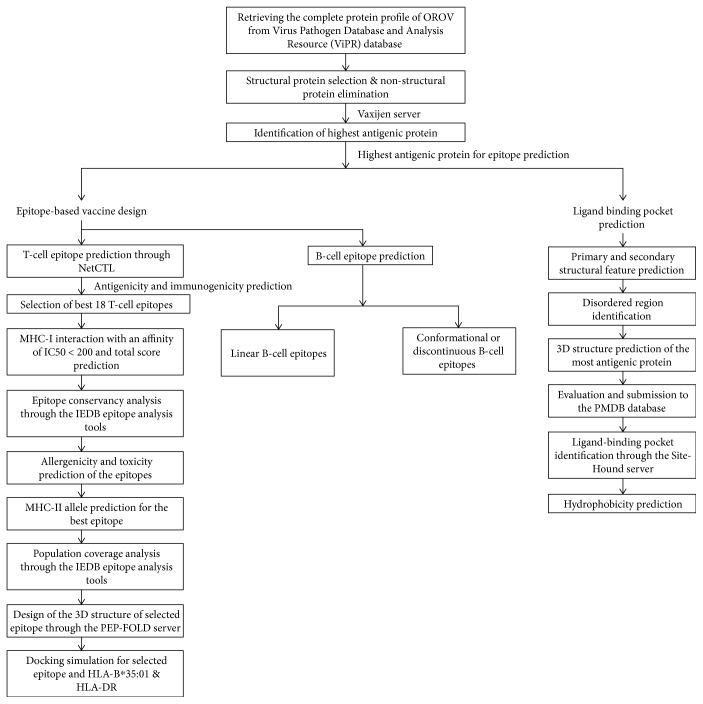
Schematic representation of epitope-based vaccine design and ligand-binding pocket prediction against the OROV polyprotein.

The flow chart representing the overall procedures of epitope-based vaccine design and ligand-binding pocket prediction for OROV polyprotein and is illustrated in Figure 1.

### 2.1. Retrieval of the Target Sequence

The FASTA formatted amino acid sequences of OROV were retrieved from the Virus Pathogen Database and Analysis Resource (ViPR) (http://www.viprbrc.org/), an integrated, powerful resource for several virus families and their respective species [18]. This database is integrated with the National Center for Biotechnology Information (NCBI) as well as UniProtKB database and helps to download the FASTA formatted sequence from both GenBank and UniProtKB. In this study, the nonstructural proteins (NSPs) were excluded from the total proteome of OROV. So, a total available 21 polymerase, 6 glycoprotein, 39 nucleocapsids, 20 polyproteins, and 46 nucleoprotein sequences were primarily selected for antigenicity prediction.

### 2.2. Highest Antigenic Protein Identification

To uncover the highest antigenic protein, the FASTA formatted amino acid sequences of total structural proteins were submitted to the VaxiJen v2.0 server (http://www.ddg-pharmfac.net/vaxijen/VaxiJen/VaxiJen.html) for antigenicity prediction [19]. This approach led to the selction of the highly antigenic protein for further analysis.

### 2.3. T-Cell Epitope Identification

The T-cell epitopes are typically peptide fragments which are immunodominant and can elicit specific immune responses, important for epitope-based peptide vaccine design. Due to the importance of T-cell epitopes, the NetCTL 1.2 server was used for the prediction of cytotoxic T-lymphocyte (CTL) epitopes derived from the protein sequence. The NetCTL 1.2 server (http://www.cbs.dtu.dk/services/NetCTL/) can predict human CTL epitopes in any given protein. The NetCTL 1.2 server can predict CD8^+^ T-cell epitopes for 12 supertypes such as A1, A2, A3, A24, A26, B7, B8, B27, B39, B44, B58, and B62 taking into account the proteasomal C terminal cleavage, MHC class I binding, and TAP transport efficiency. MHC class I binding, proteasomal C terminal cleavage, and TAP transport efficiency are predicted using artificial neural networks and the weight matrix, respectively [20, 21]. We identified epitopes for all the 12 supertypes. In this study, the threshold value for epitope identification was set at 1.25 which has a sensitivity and specificity of 0.54 and 0.993, respectively. The weight on C terminal cleavage and TAP transport efficiency were used as default parameters. Initially, 128 epitopes were selected based on the highest combined score, but the final selection completed after the antigenicity and immunogenicity prediction of the epitopes through the VaxiJen v2.0 server and IEDB server, respectively.

For the identification of both frequently and nonfrequently occurring MHC-I-binding alleles, the epitopes were analyzed by the stabilized matrix base method (SMM) in the IEDB analysis tool (http://tools.iedb.org/mhci/) [22, 23]. The amino acid length of peptide 9.0 and the IC50 value less than 200 were selected as parameters for the identification of MHC-I-binding alleles. Here, peptides with IC50 values < 50 nM are considered as high affinity, <500 nM intermediate affinity, and <5000 nM low affinity. So, the lower IC50 value indicates higher affinity. IEDB is a resourceful server, and this server was also used for the prediction of processing score, proteasomal cleavage, TAP score, and the MHC-I-binding score of the selected epitopes and their respective alleles using the SMM [24, 25]. The SMM-align method was used for the prediction of MHC-II-binding alleles of the best candidate epitope [26].

### 2.4. Epitope Conservancy and Immunogenicity Prediction

For epitope conservancy and immunogenicity prediction, the conservancy (http://tools.iedb.org/conservancy/) and immunogenicity (http://tools.iedb.org/immunogenicity/) prediction tools of IEDB were used [26, 27]. The conservancy indicates the specific portion of a protein sequence that restrains the epitope and shows availability with a specific level of identity. More immunogenic peptides are superior to the less immunogenic peptides and considered as effective T-cell epitopes. So, the epitope with high immunogenicity was selected for further analysis.

### 2.5. Prediction of Population Coverage

We used the IEDB population coverage tool (http://tools.iedb.org/population/) for the analysis of the population coverage of the predicted epitopes and their respective MHC HLA-binding alleles [28].

### 2.6. Allergenicity and Toxicity Extrapolation

Allergenicity was anticipated through AllerTOP v. 2.0 (http://www.ddg-pharmfac.net/AllerTOP/) [28] and AllergenFP 1.0 (http://www.ddg-pharmfac.net/AllergenFP/) [29]. AllergenFP 1.0 has been developed based on a novel alignment-free descriptor-based fingerprint approach. AllergenFP 1.0 can identify both allergens and nonallergens with 87.9% accuracy [29]. On the other hand, AllerTOP v. 2.0 is a robust and strong complimentary approach which has been developed based on the method, *k*-nearest neighbours (*k*NN), to classify allergens and nonallergens. The comparison among different allergen prediction servers designates that AllerTOP v. 2.0 is the best-performing method with 88.7% accuracy which is followed by AllergenFP 1.0 (87.9%) [28]. The toxicity of the peptides was predicted through the ToxinPred (http://www.imtech.res.in/raghava/toxinpred/) web server [30]. This method was developed based on the machine learning technique and quantitative matrix using different properties of peptides.

### 2.7. Three-Dimensional Structure Design of the Best Epitope

After analyzing the conservancy of epitopes, combined score, antigenicity, immunogenicity, nontoxicity, and the availability of alleles, we identified the candidate epitopes ^1043^TSSWGCEEY^1051^ and ^4^LAIDTGCLY^12^ and submitted to the PEP-FOLD peptide structure prediction server (http://bioserv.rpbs.univ-paris-diderot.fr/services/PEP-FOLD/) at the Ressource Parisienne en Bioinformatique Structurale Mobyle Portal [31, 32]. We used the best model for analysis of the interaction with MHC-I- and MHC-II-binding alleles.

### 2.8. Docking Simulation Study

To know the interaction between binding alleles and predicted epitope, the molecular docking simulation study was performed using AutoDockTools [33] and AutoDock Vina software [34]. For this purpose, the crystal structure of the HLA-B^∗^35:01 protein molecule named 3LKR was retrieved from the RCSB Protein Data Bank (PDB) as the PDB format [35]. However, the predicted crystal structure was in a complex form with protein and an epitope. So, the Discovery Studio (version 16.1.0.15350) was used to simplify the complex structure. After the separation, the protein molecule and the peptide (epitope) were then converted to PDBQT format through AutoDockTools. For the identification of binding energy at the binding groove of HLA-B^∗^35:01 with an epitope, the space box center was set at 4.496, −12.428, and −35.544 Å in the *X*, *Y*, and *Z* axes, respectively. The size was set at 30, 24, and 18 Å in the *X*, *Y*, and *Z* dimensions, respectively, and these analyses were done at a 0.964 Å spacing parameter. Finally, the docking simulation was performed by using AutoDock Vina.

Another docking study was performed as a control for the critical evaluation and scientific acceptance of our docking study. The docking method and parameters were set just like the previously mentioned parameters so that we can easily compare between sample and control. In the control section, the docking simulation was completed between influenza NP418 epitope from the 2009 strain and the HLA-B^∗^35:01 protein molecule. The docking parameters for HLA-DR and the selected epitope were set at 20.066, 31.590, and 29.302 Å in the *X*, *Y*, and *Z* axes, respectively, and the size was set at 24, 36, and 18 Å in the *X*, *Y*, and *Z* dimensions, respectively. This docking was completed at a 0.769 Å spacing parameter.

### 2.9. Prediction of B-Cell Epitopes

B-cell epitope identification is the vital step for epitope-based peptide vaccine design. So, the B-cell epitopes were identified from the highest antigenic protein through the online BepiPred-2.0 (http://www.cbs.dtu.dk/services/BepiPred/) [36] and LBtope methods (http://crdd.osdd.net/raghava//lbtope/) [37]. BepiPred-2.0 has been developed based on the random forest algorithm which was trained on epitopes annotated from antigen-antibody protein structures. BepiPred-2.0 can be considered as the best and up-to-date B-cell epitope prediction method because it shows significant results both on epitope data derived from a large collection of linear epitopes taken from the IEDB database and on crystallography-derived structural epitope data [36]. LBtope is another powerful tool for linear B-cell epitope prediction which has been developed based on the experimentally validated non B-cell epitopes obtained from the IEDB database [37]. The Lbtope_confirm dataset, the amino acid length 15-mer, and the scoring threshold of 60% were selected as the parameters for the prediction of linear B-cell epitopes in the LBtope server.

The conformational or discontinuous B-cell epitopes were predicted by using the IEDB conformational B-cell prediction tool ElliPro (http://tools.iedb.org/ellipro/) [38]. The conformational B-cell epitope is the sequence of amino acid or subunits which comprise an antigen and can come in direct interaction with a receptor of the immune system. However, ElliPro is the most comprehensive method because it can predict both the linear and conformational epitopes based on a protein 3D structure and showed the score as a PI (protrusion index) value [38]. The parameters for conformational epitope prediction were set at 0.8 and 7 Å for a minimum score and maximum distance (Angstrom), respectively.

### 2.10. Ligand-Binding Pocket Prediction

For the assessment of various physiological and physicochemical properties of the selected most antigenic OROV polyprotein, different Bioinformatics tools such as ExPASy ProtParam tools (https://web.expasy.org/protparam/) and the PSIPRED (http://bioinf.cs.ucl.ac.uk/psipred/) server were used. ExPASy ProtParam [39] was used for the prediction of amino acid composition, molecular weight, extinction coefficient, isoelectric point (pI), instability index, aliphatic index, and grand average hydropathicity (GRAVY) value. The secondary structural properties such as alpha helix, extended strands, and random coils were predicted through the PSIPRED server [40]. DISOPRED3, an integrated tool of the PSIPRED server, was used for the enumeration of disordered regions or disease-causing regions [41]. The I-TASSER server (https://zhanglab.ccmb.med.umich.edu/I-TASSER/), a unified platform for automated protein structure prediction [42, 43], was used for the design of the three-dimensional (3D) structure of the OROV polyprotein. The predicted best model was refined two times by using the ModRefiner Server (https://zhanglab.ccmb.med.umich.edu/ModRefiner/) [44] and further refined by the Swiss-PdbViewer version 4.1.0 [45] with a harmonic constraint of 20 kJ/mol/Å^2^. This energy score was applied to all protein atoms, using the steepest descent and conjugate gradient method to reduce the bad contacts between protein atoms and structural water molecules. Computations were done in vacuo with the GROMOS96 43B1 parameter set, without reaction field implementation of Swiss-PdbViewer. To validate the protein model, we used different evaluation tools such as ProSA [46], QMEAN [47], and the ProQ server [48]. The stereochemical property of the predicted protein model was checked using PROCHECK through the PDBsum server [49, 50]. Finally, the refined and evaluated protein structure was submitted to the PMDB database (https://bioinformatics.cineca.it/PMDB/) [51] for a unique accession identity number. The hydrophobicity properties of the protein were calculated through the Discovery Studio (version 16.1.0.15350).

The active site or ligand-binding site analyses make a clear perception of the molecular docking simulation study. The binding pockets of the OROV polyprotein structure were explored by the SiteHound-web (http://scbx.mssm.edu/sitehound/sitehound-web/Input.html) [52] using default parameters. This server identifies ligand-binding sites by computing the interactions between a chemical probe and a protein structure.

## 3. Results

### 3.1. Selection of the Highest Antigenic Protein

The query for Oropouche virus structural and nonstructural proteins resulted in a total of 208 hits in the ViPR database. All the structural proteins were evaluated by the VaxiJen v2.0 server, which predicted an overall score for each protein sequence and denoting the protein sequence as antigen or nonantigen. The protein sequence with the GenBank id ALB07207.1 exhibited the highest antigenic score 0.5124 among all the structural proteins. The protein itself is the OROV polyprotein containing 1420 amino acid residues, and this polyprotein was used for further analyses.

### 3.2. T-Cell Epitope Identification

The NetCTL 1.2 web server was used for the prediction of CD8^+^ T-cell epitopes, and we considered the combined score for the epitope selection. In this study, we unraveled 128 epitopes in total, which achieved the selected threshold value 1.25 (Table S1). The predicted T-cell epitopes were further evaluated by the VaxiJen server and the IEDB immunogenicity prediction tool. It has been repeated twice due to the loss of antigenicity property perceived in some T-cell epitopes. This repetition results facilitated to get a good rate of T-cell epitope prediction. Herein, among 128 primarily selected T-cell epitopes, 59 epitopes were under the threshold value 0.00 on the VaxiJen server, whereas 56 epitopes contained the immunogenicity value less than 0.00 (Table S1). So, the results revealed that all epitopes with the highest combined score could not be the best epitopes. Among 128 epitopes, we selected 37 epitopes which have positive antigenicity and immunogenicity scores, so that these highly antigenic epitopes can interact to the MHC alleles with high affinity and can create an effective immune response (Table S2). Finally, we identified 18 epitopes which contained more than >70% conservancy score, because the conservancy score is an important issue during the development of an effective vaccine. Epitope conservancy among the different strains of the selected protein can provide immunization effectively. So, the higher conservancy of an epitope ensures the better target for improved vaccine design. The selected epitopes, conservancy score, and their position are shown in Table 1. In this study, 18 epitope conservancy analysis results revealed that the epitopes TSSWGCEEY, CSMCGLIHY, and LAIDTGCLY are 100.0% conserved, whereas the rest of the other 15 epitopes are conserved from 71.43% (RQHRGCIRF, YNVAWRTYL, and YLKNHNIDL) to 95.24% (CEEYGCLAI). However, among 18 T-cell epitopes, seven epitopes, including LIHYRPGLK, YLKNHNIDL, CEEYGCLAI, VPRYHSIDV, IPAISGLGV, AISGLGVGY, and RQHRGCIRF, have the VaxiJen score of more than 1 (>1) which indicates the high antigenicity nature of these epitopes (Table 1). The immunogenicity score of the epitope can be a good criterion for the selection of the best epitope. The higher score designates a greater probability of eliciting an effective immune response. Herein, the epitopes RQHRGCIRF (0.18739), LEPIIGDKL (0.18454), and NLPHVVPRY (0.12889) are less immunogenic than the epitopes IFAAIIFAL (0.42861), SLIEICITL (0.3578), YNVAWRTYL (0.34779), IIFALIITK (0.32685), and TSSWGCEEY (0.29444), among 18 epitopes (Table S2).

The selected 18 T-cell epitopes were found to be recognized by the significant MHC class-I molecule such as HLA-A, HLA-B, and HLA-C according to IEDB MHC class 1-binding analysis resource. We selected humans as MHC source species and the SMM method for the prediction of a distinct set of MHC HLA alleles for the humans. This tool gives an output result for HLA-binding affinity of the epitopes in the IC50 nM unit. A lower IC50 value indicates higher binding affinity of the epitopes with the MHC class I molecule. So, in this study, we chose IC50 values less than 200 nM (IC50 < 200) for ensuring higher affinity (Table 1). For MHC-I processing, the analysis tool of the IEDB generates an overall score for each epitope's intrinsic potential of being a T-cell epitope based on proteasomal processing, TAP transport, and MHC-I-binding efficiency. Before being presented to the T-cells on the plasma membrane of the cell, the protein is degraded into small peptides in the proteasome by the cytosolic proteases and MHC-1 forms a complex with the peptides as well. Then, the MHC-I peptide complex is transported to the endoplasmic reticulum (ER) through heat shock proteins and transport-associated proteins (TAP). However, the higher the total score of the epitopes with the HLA alleles ensures the presentation to the T-cell and a successful immune response critically depends on it. The epitopes with their respective alleles and total scores are summarized in Table 1 and Table S2. In this in silico analysis, the epitopes “CEEYGCLAI” and “RQHRGCIRF” were found as toxic to humans.

Effective immune response not only depends on the successful recognition of epitopes by HLA molecules with significant affinity but also depends on the antigenicity and immunogenicity score. So, the epitopes that were recognized by the considerable number of HLA alleles and contained the highest immunogenicity, antigenicity value, and nontoxic to human were considered as the potential epitope to induce a strong immune response. So, we selected two 100.0% conserved 9-mer epitopes “TSSWGCEEY” and “LAIDTGCLY” for docking purposes. Here, the epitope “TSSWGCEEY” showed the highest affinity for 11 MHC-1 molecules, including HLA-B^∗^27:20 (8.37), HLA-C^∗^03:03 (14.05), HLA-A^∗^32:07 (14.19), HLA-A^∗^68:23 (22.63), HLA-C^∗^12:03 (23.03), HLA-B^∗^40:13 (65.91), HLA-A^∗^32:15 (101.22), HLA-C^∗^07:01 (104.75), HLA-B^∗^15:02 (126.83), HLA-B^∗^15:17 (136.68), and HLA-B^∗^35:01 (180.13), whereas the epitope “LAIDTGCLY” exhibited the affinity with HLA-C^∗^03:03 (8.35), HLA-C^∗^12:03 (9.53), HLA-B^∗^15:17 (10.93), HLA-A^∗^68:23 (17.90), HLA-B^∗^35:01 (25.92), HLA-A^∗^32:07 (28.58), HLA-A^∗^29:02 (73.92), HLA-B^∗^15:01 (122.74), HLA-B^∗^15:03 (136.49), HLA-B^∗^15:02 (160.04), and HLA-B^∗^40:13 (169.80) (Table 1). So, 7 MHC-I alleles HLA-C^∗^03:03, HLA-A^∗^32:07, HLA-C^∗^12:03, HLA-B^∗^40:13, HLA-B^∗^15:02, HLA-B^∗^15:17, and HLA-B^∗^35:01 were found as common in both “TSSWGCEEY” and “LAIDTGCLY.” However, we selected HLA-B^∗^35:01 for docking purposes due to the availability of this allele in the PDB database. After that, we further used the epitopes “TSSWGCEEY” and “LAIDTGCLY” for the prediction of MHC-II alleles and their respective peptide or CD4^+^ T-cell epitope. The results showed that the epitope “TSSWGCEEY” and respective 15-mer peptides “KEHTSSWGCEEYGCL” and “EHTSSWGCEEYGCLA” have low affinity with the predicted MHC-II alleles (Table 2). On the other hand, the epitope “LAIDTGCLY” was found as the core sequence of 31 15-mer peptides or CD4^+^ T-cell epitopes in the range of the IC50 value 1–3000 nM. Among these 31 peptides, ten peptides exhibited a strong affinity (IC50 value 194 to 611 nM) with HLA-DR alleles, HLA-DRB3^∗^01:01, HLA-DRB1^∗^03:01, and HLA-DRB1^∗^12:01 (Table 2). So, HLA-DR alleles could be the best-binding grooves for the predicted peptides.

### 3.3. Analysis of Population Coverage and Allergenicity

**Figure 2:**
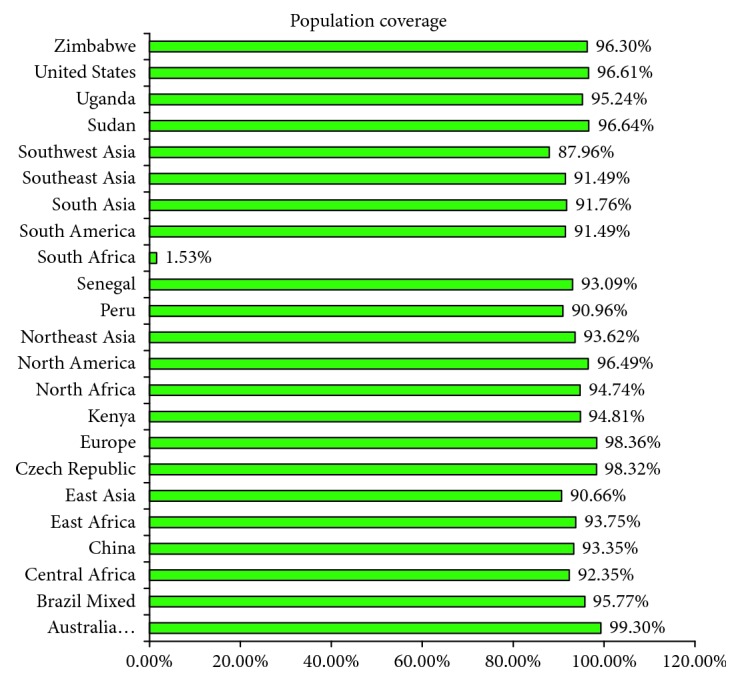
Population coverage of the epitopes and their respective HLA alleles of the OROV polyprotein. The epitopes with different HLA-binding specificities increase the population coverage as the several HLA types are expressed at radically different frequencies in various ethnicities.

MHC HLA allele distribution differs among diverse geographic regions and ethnic groups around the world. Therefore, population coverage must be taken into consideration during the design of an effective vaccine. In this study, for the population coverage, identified MHC-I-binding alleles of 18 epitopes were considered. A significant population coverage was found for the selected 18 epitopes in different geographic regions of the world (Figure 2 and Table S3). These epitopes and their HLA-alleles cover 96.44% of the world population. The highest population coverage was found in the European country Finland (99.69%) which was closely followed by Ireland, Germany, England, Poland, France, Italy, and Sweden with a population coverage of 99.51%, 99.19%, 98.66%, 98.60%, 98.40%, 97.27%, and 96.99%, respectively. The lowest population coverage was found in South Africa (1.53%). The Oropouche fever was first found in South America, especially in Brazil, and several outbreaks have been recorded in this country. So, the population coverage prediction in Brazil is essential for vaccine design. The cumulative population coverage in Brazil is 93.92%, but it is found as 95.77% in mixed Brazilian people (Table S3). However, another South American country Chile has 92.79% population coverage. It varies in the North American country, especially in the United States people, and mostly covers the United States Caucasian race (98.96%) and United States African American (96.18%), United States Hispanic (95.86%), United States Mestizo (95.64%), United States Asian (94.58%), United States Polynesian (93.86%), and United States Amerindian (90.99%) people (Table S3). The population coverage in West Africa is 91.97%, whereas the North African country Morocco and Sudan covers 96.69% and 96.64%, respectively, also followed by the East African country Zambia (97.75%) and Kenya (94.81%). However, the Oceanian country, Australia (Caucasian race), covers about 99.30% populations for the selected 18 epitopes and their respective alleles (Figure 2).

Before vaccine design, the allergenicity prediction is a key step, because most vaccines transfer the immune response to the allergic reaction by initiating immunoglobulin E and Type II T-helper cells [53]. In that case, we predicted the allergenicity of the selected epitope through the AllergenFP 1.0 server. The server identified the epitopes, TSSWGCEEY, CSMCGLIHY, VPRYHSIDV, YNVAWRTYL, LEPIIGDKL, NSGPYNVAW, and AISGLGVGY as nonallergen and the rest of the other epitopes as a potential allergen to humans (Table 1). On the contrary, AllerTOP v. 2.0 showed the allergenicity nature for the 100.0% conserved epitope TSSWGCEEY and 80.95% conserved epitope VPRYHSIDV. However, the results were found as the same for the rest of the other five epitopes (CSMCGLIHY, YNVAWRTYL, LEPIIGDKL, NSGPYNVAW, and AISGLGVGY) in both AllergenFP 1.0 and AllerTOP v. 2.0 servers (data not shown). The predicted three-dimensional structures of the T-cell epitopes were deposited to the Protein Model Database (PMDB). As a result, the database provided a unique accession number for each of the submissions of the epitope. The structure of the epitopes could be identified by using the accession number of PM0081248 and PM0081251 for T-cell epitopes TSSWGCEEY and LAIDTGCLY, respectively, in the PMDB database.

### 3.4. Docking Simulation Study

**Figure 3:**
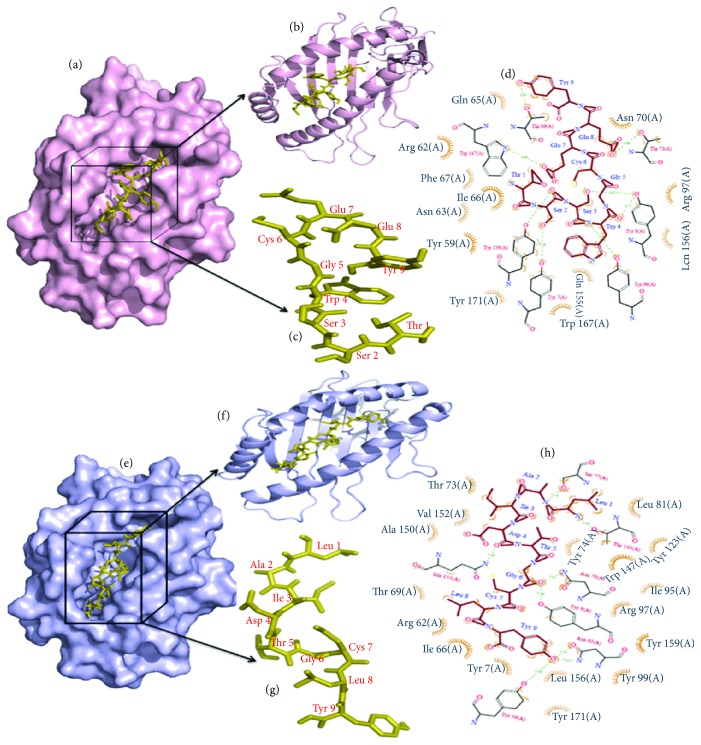
Docking simulation study (sample). (a) The docking results as the surface structure of the MHC-I HLA allele “HLA-B^∗^35:01” (tint light pink color) and the sticky form of epitope “TSSWGCEEY” (orange olive color). The black color box represents the position of epitope in the surface structure. (b) Epitope in the cartoon structure. (c) Sticky form of epitope “TSSWGCEEY” (orange olive color) with residue's position. (d) Binding interaction of the epitope residues and the HLA-B^∗^35:01 residues through the hydrogen bonds. Hydrogen bonds and the distances are shown in green color. (e) The docking results as the surface structure of the MHC-I HLA allele “HLA-B^∗^35:01” (tint light blue color) and the sticky form of epitope “LAIDTGCLY” (orange olive color). The black color box represents the position of epitope in the surface structure. (f) Epitope LAIDTGCLY in the cartoon structure. (g) Sticky form of epitope “LAIDTGCLY” (orange olive color) with residue's position. (h) Binding interaction of the epitope residues and the HLA-B^∗^35:01 residues through the hydrogen bonds. Hydrogen bonds and the distances are shown in green color.

The molecular docking simulation study anticipated nine (9) potential binding affinities between the HLA-B^∗^35:01 and epitope TSSWGCEEY. At the same time, it was also found in the interaction between HLA-B^∗^35:01 and epitope LAIDTGCLY. The finest model was selected by higher binding affinity. The best interrelating model of epitope TSSWGCEEY and the HLA molecule contained a binding affinity of −8.0 kcal/mol. On the other hand, the epitope LAIDTGCLY and HLA molecule contained a binding affinity of −8.4 kcal/mol. The epitope position in the cartoon and surface structure and the binding interactions are shown in Figure 3. The epitope and the HLA molecule interacted by the hydrogen bonds, and we found nine hydrogen bonds after the docking simulation between HLA-B^∗^35:01 and epitope TSSWGCEEY. In this study, the amino acid residues, namely, Tyr-7, Tyr-9, Tyr-99, Tyr-159, Trp-147, Thr-69, and Thr-73, acted as the hydrogen bond donors or acceptors with the epitope residues (Figure 3). Herein, eight hydrogen bonds, 89 nonbonded contacts, and the interacting residues, Thr-143, Ser-77, Gln-155, Tyr-9, Asn-70, and Tyr-59, were found for HLA-B^∗^35:01 and epitope LAIDTGCLY.

The cartoon and surface structures were prepared and visualized by the PyMOL molecular graphics system.

In the control section, the docking between HLA-B^∗^35:01 and the influenza NP418 epitope (LPFERATVM) from the 2009 strain was performed. The best affinity or binding energy between these two molecules was found −8.3 kcal/mol, which is very close to the sample. The amino acid residues, namely, Tyr-7, Tyr-9, Tyr-99, Tyr-159, Tyr-171, Ser-77, and Gln-155, are involved in the interaction with the epitope residues (Figure S1). So, the results revealed that Tyr-7, Tyr-9, Tyr-99, and Tyr-159 are conserved residues in the docking simulation of the sample (TSSWGCEEY) and control and ensuring the accuracy of the binding affinity. Besides, Tyr-9, Ser-77, and Gln-155 residues were found as common residues in the docking simulation of the sample LAIDTGCLY (binding affinity of −8.4 kcal/mol) and control which also ensures the accuracy and the acceptance of the docking simulation.

**Figure 4:**
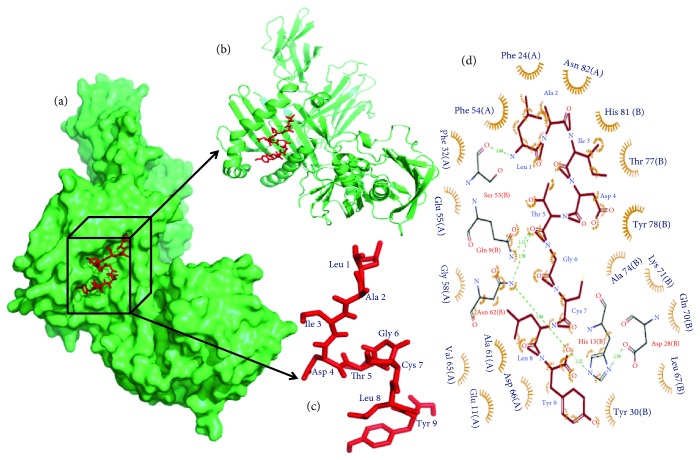
Docking simulation study. (a) The docking results as the surface structure of the MHC-II (HLA-DR) (lime green color) and the sticky form of epitope “LAIDTGCLY” (red color). Black color box represents the position of epitope in the surface structure. (b) Epitope in the cartoon structure. (c) Sticky form of epitope “LAIDTGCLY” (red color) with residue position. (d) Binding interaction of the epitope residues and the HLA-DR residues through the hydrogen bonds. Hydrogen bonds and the distances are shown in green color.

The epitope (peptide) binding to the MHC class II HLA-DR molecule is important for the proper functioning of a vaccine candidate. So, we predicted the alleles for our selected epitope and performed a docking study between those molecules. Herein, we found the best binding affinity −7.9 kcal/mol for the docking study among the predicted nine binding affinity results. This result indicates a good affinity between HLA-DR and the epitope LAIDTGCLY (Figure 4). During the analysis of the interaction between HLA-DR and LAIDTGCLY, we found five hydrogen bonds and 125 nonbonded contacts in the combined structure. However, the amino acid residues, namely, Ser-53, Gln-9, Asn-62 (2 times), and His-13, are involved in the interaction with the epitope residues (Figure 4).

### 3.5. Analysis of Linear and Conformational B-Cell Epitopes

A B-cell epitope is a precise portion of an antigen recognized by either a specific B-cell receptor or the elicited antibody in a humoral response [23, 54]. The B-cell epitopes are divided into two main categories such as continuous or linear B-cell epitope and discontinuous or conformational B-cell epitope. It has been found that most of the B-cell epitopes are conformational epitopes, and the amount of this epitope is more than 90% [55].

The prediction of B-cell epitopes from the antigenic protein is the major step of epitope-based vaccine design. Thus, to get essential B-cell epitope candidates in the polyprotein of OROV, we performed an in silico analysis through the web server BepiPred-2.0 and LBtope.

Based on the BepiPred-2.0 method, a total of 31 linear B-cell epitopes with varied length were identified from the polyprotein of OROV. Among all these predicted epitopes, 8 B-cell epitopes ^81^VKDWSECNPVLDKL^94^, ^334^AGERMYKLEELADD^347^, ^504^YIMGDTCVSKSELYSDLLSKNLITEYDKKYFEPDTVN^540^, ^875^CSEQRFPIHKNWFKKCNWDKTHKEFTTMRQINYNDITSYRKAIES^919^, ^922^GTDLMTHHYKPTKNLPHVVPRYH^944^, ^1095^ETYCNDMDILEPIIGD^1110^, ^1186^FNDNYQSCT^1194^, and ^1231^DINYKIFTKSV^1241^ were nonantigenic as predicted by the VaxiJen v2.0 server. These epitopes had 5.56% to 55.56% conservancy level, except FNDNYQSCT (100% conservancy score), among the specified polyprotein sequences (Table 3). So, these epitopes could not be the vaccine candidates. On the other hand, the epitopes ^17^HPLSTSQIGDRC^28^ (94.44%), ^35^FKEMNLSVGL^44^ (100%), ^282^SCTEALKVHRMGKDCLGYKS^301^ (100%), ^566^ECEMLSLKKNSGPYN^580^ (100%), ^594^LCSRHNY^600^ (100%), ^1026^PEKIPAKEGWLTFSKEHTSSW^1046^ (100%), and ^1119^TQTNQLP^1125^ (100%) can be considered as vaccine candidates due to their antigenicity and high conservancy score (Table 3). After the prediction of allergenicity (AllerTOP 2.0 and AllergenFP v. 1.0) and toxicity, it can be said that the epitopes ^17^HPLSTSQIGDRC^28^, ^1026^PEKIPAKEGWLTFSKEHTSSW^1046^, and ^788^SHCNLEFTAITADKIMSL^805^ could be the potential linear B-cell epitopes for vaccine design (Table 3).

During the prediction by LBtope, a total of 19 linear B-cell epitopes were found from the OROV polyprotein. The epitopes ^13^IAYGHPLSTSQI^24^, ^110^PRMYTCRAACDI^121^, ^432^SSGFEYEVFLKDMH^445^, ^519^DLLSKNLITEYDKKYFEPDTVNDQFNKIEFAQD^551^, ^619^DFNNEIETYYKANAAAYRSDFYTI^642^, ^706^TRSAQHSIMTN^716^, ^848^CGDKYYYSDGKEHAKDGS^865^, and ^1089^CITLPHETYCNDMDILEPII^1108^ are nonantigenic among the 19 epitopes due to their low antigenicity score in the VaxiJen server (Table 4). On the contrary, the epitopes ^889^KCNWDKT^895^, ^928^HHYKPTKNLPHVVPRYH^944^, ^1273^CHCKTNCED^1281^, and ^1327^NQKIDLSQL^1335^ were found as antigenic and 100% conserved among all the predicted epitopes. However, the epitopes ^889^KCNWDKT^895^ and ^1273^CHCKTNCED^1281^ could not be the potential vaccine candidates because these two B-cell epitopes were identified as toxic to the human (Table 4).

After the similarity search between BepiPred-2.0 and LBtope predicted B-cell epitopes, we identified 18 sequences as most conserved B-cell epitopes. However, 7 B-cell epitopes were found as nonantigenic and less conserved (5.56% to 38.89%), except the epitopes ^1095^ETYCNDMDILEPII^1108^ (100%) and ^1188^DNYQSCT^1194^ (100%) (Table 5). The predicted epitope ^928^HHYKPTKNLPHVVPRYH^944^ is 100% conserved, which is also followed by ^1327^NQKIDLSQL^1335^ (100%), ^889^KCNWDKT^895^ (100%), and ^17^HPLSTSQI^24^ (94.44%). The analysis of antigenicity, conservancy, toxicity, and allergenicity of B-cell epitopes revealed that the epitope ^928^HHYKPTKNLPHVVPRYH^944^could be the most potential B-cell epitope candidate for peptide-based vaccine design because this epitope is antigenic, 100% conserved, nontoxic, and nonallergenic to the human. The exact position and length of the predicted B-cell epitopes are shown in Table 5.

**Figure 5:**
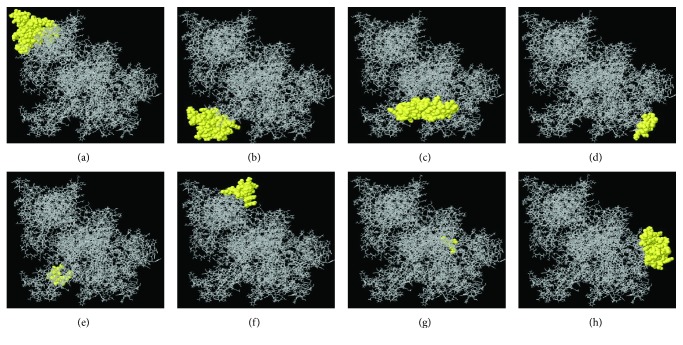
Three-dimensional representation of conformational or discontinuous epitopes (a–h) of the highest antigenic polyprotein of OROV. The epitopes are represented by yellow surface, and the bulk of the polyprotein is represented in grey sticks.

In this study, a total of 8 discontinuous or conformational B-cell epitopes were predicted using the ElliPro tool of IEDB. The epitopes having a protrusion index (PI) score above 0.8 were taken into consideration. The PI value of the 8 predicted epitopes ranged from 0.837 to 0.906. The residues with higher scores indicate better solvent accessibility. Herein, the highest probability of a discontinuous epitope was calculated at 90.6% (PI score: 0.906) and is shown in Figure 5(a). Conformational epitopes and their individual residues, residue position, length, and the scores are shown in Table 6, whereas the positions of epitopes on 3D structures are exhibited in Figures 5(a)–5(h).

### 3.6. Ligand-Binding Pocket Analysis

**Figure 6:**
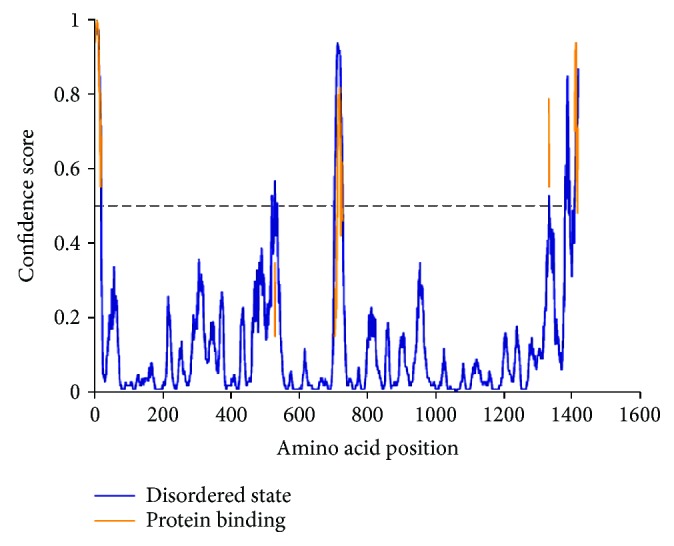
Disordered regions of the OROV polyprotein. Here, amino acids in the input sequence are considered disordered when the blue line is above the grey dashed line, that is, the confidence score is higher than 0.5. The orange line shows the confidence of disordered protein-binding residue predictions.

The physicochemical properties of the OROV polyprotein were found as 8.01 isoelectric points, 37.22 instability index value, 88.99 aliphatic index values, and −0.138 GRAVY value. The other features are shown in Table 7. These results revealed that the protein is naturally mildly basic, stable, and hydrophilic. The secondary structural features showed that the random coils with 45.99% is dominated by alpha helix (31.62%) and extended strands (22.39%) (Figure S2). The unstructured or disordered regions can play an important role in the catalytic activity of the protein that was predicted by the DISOPRED3 server. The disordered regions were found in the ^528^EY^529^, ^535^E, ^705^STRSAQHS^712^, ^723^PF^724^, and ^727^YS^728^ positions. The disordered regions and their confidence score are shown in Figure 6.

**Figure 7:**
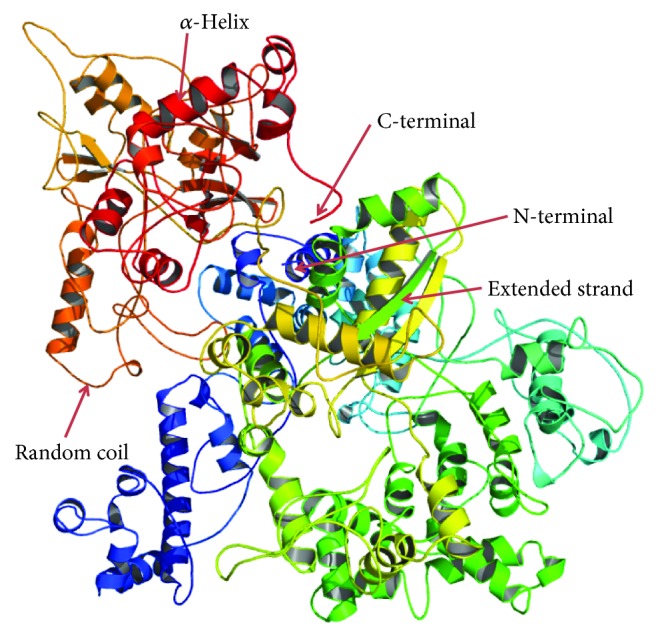
Three-dimensional structure of the OROV polyprotein. This protein model was predicted by the I-TASSER server and visualized by the PyMOL molecular graphics system.

The 3D structure of the OROV polyprotein is not available in the PDB database. So, a 3D structure of the OROV polyprotein was predicted by the I-TASSER server and visualized by the PyMOL tools (Figure 7). Different evaluation scores of the I-TASSER server, such as C-score (−1.02), estimated TM score (0.59 ± 0.14), estimated RMSD (12.3 ± 4.4 Å), cluster density (0.1329), and number of decoys (213), ensured the good quality of the predicted model. Energy minimization through the ModRefiner server and Swiss-PdbViewer software tools removes the bad contacts of the predicted protein model. The total force field energy of the protein structure was −36896.734 kJ/mol after energy minimization. Here, PROCHECK was used to check the stereochemical quality of the structure by considering residue geometry and overall structural geometry. The results showed 72.2% in most favored regions, 21.9% in the additional allowed region, 3.6% in the generously allowed region, and 2.3% in the disallowed region in the Ramachandran plot statistics (Figure S3). To check the potential errors of the protein 3D model, ProSA was used, and it predicts the negative *Z*-score of −6.67, which ensures the good quality of the model (Figure S4). The ProQ server showed an LG score of 3.651 (LG score > 2.5 indicates very good model) and MaxSub score of 0.500 (MaxSub > 0.5 indicates very good model), ensuring the “very good” quality of the predicted protein model. Another evaluation tool QMEAN gave different scores with an environmental profile graph for the OROV polyprotein structure (Figure S5). The refined and evaluated protein model was submitted to the PMDB database, and the server provided a unique accession id PM0081042 for the submitted model. The hydrophobicity nature greatly impacts the drug design and delivery system. The hydrophobicity plot of the OROV polyprotein is shown in Figure S6.

The ligand-binding pockets of OROV polyprotein were not reported yet. So, the SiteHound server was used to predict the top 10 ligand-binding pockets of the OROV polyprotein which ranked by energy (Table 8). The surface volume of the binding pockets was also explored in the center *x*-axis, *y*-axis, and *z*-axis. The residues which are involved in the formation of protein cavities were explored for copious binding of new ligands such as epitope. The energy was ranged from −1677.27 to −940.23 which ensures the efficiency of the predicted top 10 ligand-binding pockets. These ligand-binding pockets could be the target for molecular docking simulation in the future.

## 4. Discussion

In recent years, many diseases have emerged due to the occurrence of several outbreaks through the different types of newer viruses. So, vaccine development against these emerging diseases within a short time is very crucial to protecting the people from the rising viral attacks. Vaccines are the pharmacological products which can provide the finest cost-benefit ratio in the prevention or treatment of diseases. However, an effective vaccine progression and production are costly and can take years to be completed. So, the researchers have tried for many years to minimize the cost and time for the development of vaccines. At this time, there are different strategies available for the design and development of effective and safe new-generation vaccines based on the Bioinformatics approaches [56, 57]. The next-generation sequencing and progressive genomics and proteomics technologies have brought about a great change in computational immunology. However, the advancement of newer immunoinformatics tools has made a broader way in developing the vaccine or vaccine candidates through the satisfactory understanding of the immune response of the human body against an organism within a short time [58–60].

After recovery from an acute viral or microbial infection, a person generally achieves long-term or even lifelong immunity to the same viral disease or pathogen [61, 62]. Numerous acute viral infections exhort a protective humoral response which is characterized by the presence of a preexisting antibody. However, the duration of this preexisting antibody response differs among viral infections and can exist for a few months to many years in the host [63]. Slifka and Ahmed reported that the antibody persistence of acute viral infection in humans, such as Chikungunya, Rift Valley fever virus, dengue, yellow fever, and measles, are about 30 years, 12 years, 32 years, 75 years, and 65 years, respectively [63]. According to Tesh and Vasconcelos, OROV could not be isolated from the patient's serum during the recurrent illness, but the detectable humoral antibodies are usually found [64]. However, no significant fatalities have been stated for OROV, and the lifelong immunity ensures the recovery from this emerging virus [64].

B-cell memory consists of humoral memory (long-lived antibody-secreting plasma cells) and reactive memory (long-lived memory B-cells which can react quickly to a recurrent antigenic challenge) [61]. Mainly memory B-cells afford the potential to respond fast and with more adapted antibodies against reinfection [61, 65]. Welsh et al. concluded that memory B-cell responses are long-lived even in the absence of antigen, but these responses are controlled and influenced by other infections in the host. This memory B-cells are responsible for providing long-term protective immunity or contribute to immunopathology on infection with homologous or heterologous viruses [66]. B-cell memory is virus-specific, but the exact cellular mechanisms of virus-specific antibody response to acute viral infection remain unclear [67]. Hebeis et al. demonstrated that the activation of virus-specific memory B-cells to secrete immunoglobulin G (Ig G) is autonomous of cognate or bystander T-cell help [68]. The role of virus-specific B-cell memory has been found in mumps virus [69], human influenza virus [70], rotavirus [71, 72], and dengue virus [73].

In recent trends, the surface glycoprotein (GP) is considered as the initial focus for the development of epitope-based peptide vaccine, as they are involved in the interaction between cell receptor and virus particle, thus playing a significant role in the pathogenesis of the disease [74]. To our knowledge, the subunit or epitope/peptide-based vaccine approaches have not been used yet for the prediction of a potential vaccine against this neglected emerging OROV. However, the virus-like particle assay has been developed for this OROV that could be used as a tool to develop a potential recombinant vaccine [11, 74]. The population coverage information of the epitopes and their respective HLA alleles are crucial to being the potential vaccine candidates. The computational prediction of this HLA allele is much easier and cost-effective than the experimental one. Therefore, in this study, different types of immunoinformatics and molecular simulation tools were used to find the potential vaccine candidates present in the OROV polyprotein.

In the present study, an immunoinformatics-driven approach was incorporated to screen emergent immunogen against OROV proteome. The results revealed that the OROV polyprotein was the most antigenic protein with the highest antigenic scores (0.5124) compared with other viral proteins of OROV. To our knowledge, no immunoinformatics study for the OROV polyprotein has been performed yet for the identification of a potential vaccine target. Although we found the polyprotein as a potential antigenic protein, another OROV protein may be the source of vaccine candidate identification. However, we identified the potential T-cell epitopes from the most antigenic polyprotein, as they play a key role in the creation of a defensive immune response against different pathogenic infections [75]. Various successful studies have been performed for the epitope-based peptide vaccine design against West Nile virus [76], Zika virus [77], dengue virus [78], Chikungunya virus [79], Rift valley fever virus [80], shigellosis [81], and so on. Primarily, we identified 146 epitopes in total using 12 supertypes (A1, A2, A3, A24, A26, B7, B8, B27, B39, B44, B58, and B62) and selected 128 epitopes based on the highest combined score. Finally, we identified 18 epitopes by maintaining some critical criteria such as antigenicity, immunogenicity, and most important conservancy scores of the epitopes (Table 1). We gave priority to the higher conservancy score of the epitopes because higher conservancy ensures the extensive protection across numerous strains or even species. Our selected 18 T-cell epitopes were 70.0% to 100.0% conserved which could ensure the acceptance of these predicted epitopes and indicate them as a potential vaccine candidate. Hossain et al. showed 65.66% conserved epitope “TLKNLNDNY” as a potential vaccine candidate for universal vaccine design against rotavirus [82]. On the contrary, Hasan et al. revealed 69.49% conserved epitope “YYYELYPTM” as a possible vaccine lead for vaccine design against Chikungunya virus [79]. Since our predicted 18 epitopes are highly conserved (3 epitopes—100% conserved, 1 epitope—95.24% conserved, 8 epitopes—80.95% conserved, 3 epitopes—76.19% conserved, and 3 epitopes—71.43% conserved), these epitopes could be considered as potential vaccine candidates for vaccine design against OROV. Predicted 100.0% conserved epitopes TSSWGCEEY, CSMCGLIHY, and LAIDTGCLY were found as antigenic, but the antigenicity was lower than the epitopes, CEEYGCLAI (95.24%), LIHYRPGLK (80.95%), VPRYHSIDV (80.95%), IPAISGLGV (76.19%), and YLKNHNIDL (71.43%). However, the epitope CSMCGLIHY could be the most important epitope in comparison with the rest of the other epitopes, because it is not allergenic (as predicted by both AllergenFP 1.0 and AllerTOP v. 2.0 servers) and nontoxic to the host. At present, the researchers are using different immunoinformatics approaches for the prediction of high potential CD4^+^ T-cell epitopes and CD8^+^ T-cell epitopes which can bind to different MHC-I and MHC-II molecules. de Freitas e Silva et al. identified 10 top-ranked epitopes from the proteome of *Leishmania braziliensis* using immunoinformatics tools and also synthesized and assessed in vitro to stimulate peripheral blood mononuclear cells (PBMC) from posttreated cutaneous leishmaniasis patients and found that 50% of these epitopes are immunogenic [83]. Khan et al. identified the T-cell epitopes from the *Mycobacterium tuberculosis* proteome using the in silico approaches for in vitro analyses and found T-cell responses during experimental evaluation of the identified epitopes [84]. So, the analysis clearly revealed that the immunoinformatics or *in silico* approaches could make a great contribution in vaccine lead identification for experimental study and/or the design of an effective vaccine.

The HLA alleles maintain the response to T-cell epitopes, and these alleles are extremely polymorphic in numerous ethnic populations. Therefore, MHC-I and MHC-II HLA allele specificity of T-cell epitopes should be considered as the initial criteria to induce extensive immune responses in diverse ethnic human populations [85, 86]. However, to get more population coverage, the T-cell epitope should bind with more MHC HLA alleles. So, we took the selected 18 epitopes and their respective MHC-I-binding HLA alleles for significant population coverage. The results revealed that the selected epitopes and their alleles have ideal population coverage in different geographic regions around the world. The highest population coverage was recorded at 99.69% in Finland, and those epitopes and their respective HLA-alleles cover 96.44% of the world population. The OROV outbreak occurred in the South American countries, especially in Brazil in the highest proportions. So, the vaccine candidates for this geographic region are essential to protecting individuals from OROV infection. The population coverage was found at 95.77% among the mixed Brazilian population, which indicates the acceptability of the epitopes for the OROV epidemic region. It was observed that the population coverage (1.53%) was found lower than the other countries (Figure 2). South Africa consists of different ethnic groups, but the HLA distribution in the allele frequency net database (AFND) is comparatively low which reflects the underrepresentation of this region. Tshabalala et al. reviewed that HLA-C alleles are mostly found in Caucasian South Africans, Black South Africans, Shona Zimbabweans, Tamil South Africans, and Zambians while HLA-B allele frequencies are found in Shonas and Black South Africans [87]. Cao et al. reported 9 different HLA alleles, HLA-A^∗^ 02:02, HLA-B^∗^15:03, HLA-A^∗^34:02, HLA-A^∗^36:01, HLA-B^∗^42:01, HLA-B^∗^53:01, HLA-B^∗^57:03, HLA-B^∗^58:02, and HLA-A^∗^74:01, as unique African alleles [88]. On the other hand, a very low amount of HLA class-II alleles is available in South African populations. Among all HLA class-II alleles, HLA-DRB1 alleles are available in most South Africans, while HLA-DP data were found in only Zimbabwean Shonas and Black South Africans, but no alleles were reported for DQA1 in AFND [87, 89]. So, the unavailability of our predicted HLA-alleles in the South African population could be the main cause of the lowest population coverage in South Africa. The 3D structure of the epitopes TSSWGCEEY and LAIDTGCLY were designed for docking purposes and compared with a control (HLA-B^∗^35:01 and influenza NP418 epitope) to measure the docking efficiency with a specific HLA allele HLA-B^∗^35:01. The docking results showed almost similar binding affinity or energy for both the control (−8.3 kcal/mol) and the sample (−8.4 kcal/mol and −8.0 kcal/mol for the epitopes “LAIDTGCLY” and “TSSWGCEEY,” respectively) which designates the satisfactory accuracy of the predicted epitopes to form an interaction with the MHC-I allele. However, another docking was performed between the MHC-II allele and the selected epitope “LAIDTGCLY” which also exhibits the satisfactory binding affinity. So, the results indicate the epitope “LAIDTGCLY” as a novel vaccine candidate of OROV.

In this study, five linear B-cell epitopes ^17^HPLSTSQIGDRC^28^ (94.44%), ^788^SHCNLEFTAITADKIMSL^805^ (83.33%), ^928^HHYKPTKNLPHVVPRYH^944^ (100%), ^1026^PEKIPAKEGWLTFSKEHTSSW^1046^ (100%), and ^1327^NQKIDLSQL^1335^ (100%) showed the highest conservancy among diverse OROV isolates compared with other epitopes, proposing that these epitopes might be the ideal vaccine candidates based on the fact that an epitope should be as conservative as possible to provide extensive protection among different virus isolates. These epitopes were also identified as nontoxic to humans. However, the cross processing of the BepiPred-2.0 and LBtope predicted B-cell epitope data revealed that the 17-mer epitope ^928^HHYKPTKNLPHVVPRYH^944^ is the most potent B-cell epitope. Besides, the discontinuous B-cell epitopes were drawn on the polyprotein of OROV (Figure 5). All the predicted discontinuous or conformational epitopes were placed on the surface of the OROV polyprotein, representing the accessibility for the entered virus.

As recommended by the antigenicity analysis through the VaxiJen server, the polyprotein of OROV seemed to be a suitable vaccine target. The important information was found from the physicochemical properties and secondary structural features of the protein by the ExPASy ProtParam and PSIPRED servers. The protein was found to be basic, stable, and hydrophilic in nature and could sustain high temperature due to a higher aliphatic index value. The abundance of random coils in the OROV polyprotein structure ensured the higher conservancy and stability of the protein [39, 90]. This protein contained disordered regions in some specific positions. The most disordered region was positioned in the 705 to 712 (STRSAQHS) location in the protein. The predicted 3D structure of the OROV polyprotein by the I-TASSER server was acceptable. The energy minimization and evaluation results ensured the good quality of the predicted model. The evaluated model was submitted to the PMDB database for further research in the future. To support the future drug discovery and drug design, ligand-binding pockets and hydrophobicity analyses were accomplished. However, ten possible ligand-binding pockets and their respective amino acid residues were found. The results suggested that the binding residues of these pockets could be the future research prospect for effective inhibitor design against emerging OROV infection.

Peptide's half-life prediction is one of the key challenges in successful peptide vaccine design as its bioavailability depended on the extent of the half-life. So it is vital to identify the optimal half-life of the peptide for ensuring their optimal function [91] post-vaccination. Recently, a database called “PEPlife” has been developed as a repository of the half-life of peptides [92]; however, it is customary to test and measure for the peptide half-life to ensure optimal protection post-vaccination. Cytokines play an important role in elucidating humoral immune response against antiviral activity, and methods have been developed to predict the different interleukins such as IFN-*γ* [93], IL-4 [94], and IL-10 [95]. In general, protein-based vaccines have limited immunogenicity and reactogenicity compared to live-attenuated and inactivated vaccines, although protein-based vaccines have greater advantages in relation to safety and cost-effectiveness [96, 97]. An overarching strategy to achieve maximum protection against viral infections is to design a successful peptide-based vaccine following the identification of crucial epitopes via the immunoinformatic approach combined with effective adjuvant choice. Adjuvants are substances added to vaccine formulations that influence the onset, strength, and longevity of specific immune responses to antigens and induce protection against infection [96, 98]. Moreover, adjuvants receive much attention due to their ability to selectively modulate the immune response to elicit humoral and/or cell-mediated immune response [97]. A number of approved vaccine adjuvants such as aluminium salts, oil-in-water (o/w) emulsions, virosomes, liposomes, monophosphoryl lipid A, and TLR agonist are available, but at this time in silico adjuvant prediction tools are not available due to the unavailability of sufficient data and exact molecular mechanism of vaccine adjuvants.

## 5. Conclusion

This study provided valuable insights for the identification of vaccine candidates and ligand-binding pockets against the neglected and emerging OROV infection through the immunoinformatics as well as Bioinformatics approaches. Based on this strategy, the T-cell and B-cell epitopes in the polyprotein of OROV were mapped and selected as putative OROV vaccine targets. The predicted epitopes exhibited T-cell and B-cell selectivity, higher conservancy, nonallergenicity, nontoxicity, higher population coverage, and significant interaction with MHC class I allele with good affinity. Nonetheless, these findings will provide primary data for molecular docking and epitope-based peptide vaccine research in the future.

## Figures and Tables

**Table 1:** Most potential 18 T-cell epitopes with interacting MHC-I alleles, epitope conservancy score, allergenicity, and toxicity.

Epitopes	Position	Antigenicity	MHC-I alleles	Conservancy	Allergenicity	Toxicity
TSSWGCEEY	1043–1051	0.9867	HLA-B^∗^27:20, HLA-C^∗^03:03, HLA-A^∗^32:07, HLA-A^∗^68:23, HLA-C^∗^12:03, HLA-B^∗^40:13, HLA-A^∗^32:15, HLA-C^∗^07:01, HLA-B^∗^15:02, HLA-B^∗^15:17, HLA-B^∗^35:01	100.00%	No	Yes
CSMCGLIHY	48–56	0.5400	HLA-B^∗^27:20, HLA-A^∗^68:23, HLA-C^∗^12:03, HLA-A^∗^32:07, HLA-B^∗^15:17, HLA-B^∗^40:13, HLA-B^∗^15:02, HLA-B^∗^15:01, HLA-C^∗^14:02, HLA-A^∗^32:15, HLA-B^∗^15:03, HLA-A^∗^30:02	100.00%	No	Yes
LAIDTGCLY	4–12	0.9551	HLA-C^∗^03:03, HLA-C^∗^12:03, HLA-B^∗^15:17, HLA-A^∗^68:23, HLA-B^∗^35:01, HLA-A^∗^32:07, HLA-A^∗^29:02, HLA-B^∗^15:01, HLA-B^∗^15:03, HLA-B^∗^15:02, HLA-B^∗^40:13	100.00%	Yes	Yes
SLIEICITL	34–42	0.9867	HLA-A^∗^02:11, HLA-A^∗^32:07, HLA-A^∗^02:12, HLA-A^∗^02:16, HLA-A^∗^02:01, HLA-A^∗^68:23, HLA-A^∗^02:02, HLA-A^∗^02:50, HLA-B^∗^27:20, HLA-C^∗^03:03, HLA-A^∗^02:03, HLA-A^∗^02:06, HLA-C^∗^12:03, HLA-A^∗^02:17, HLA-B^∗^15:02, HLA-C^∗^07:02, HLA-C^∗^14:02, HLA-A^∗^02:19, HLA-A^∗^32:15	76.19%	Yes	Yes
IIFALIITK	12–20	0.7267	HLA-C^∗^12:03, HLA-A^∗^11:01, HLA-A^∗^32:07, HLA-B^∗^40:13, HLA-A^∗^03:01, HLA-B^∗^27:20, HLA-A^∗^68:23, HLA-A^∗^68:01, HLA-A^∗^32:15, HLA-C^∗^03:03, HLA-A^∗^30:01	80.95%	Yes	Yes
LIHYRPGLK	53–61	1.7718	HLA-A^∗^32:07, HLA-B^∗^27:20, HLA-C^∗^12:03, HLA-C^∗^03:03, HLA-A^∗^68:23, HLA-A^∗^30:01, HLA-A^∗^03:01, HLA-B^∗^40:13, HLA-A^∗^32:15	80.95%	Yes	Yes
IFAAIIFAL	8–16	0.6140	HLA-C^∗^03:03, HLA-A^∗^02:50, HLA-B^∗^27:20, HLA-A^∗^02:02, HLA-A^∗^68:23, HLA-B^∗^40:13, HLA-A^∗^32:07, HLA-A^∗^02:11, HLA-A^∗^02:06, HLA-B^∗^15:02, HLA-C^∗^14:02, HLA-B^∗^42:01, HLA-C^∗^12:03, HLA-C^∗^07:02, HLA-A^∗^32:15, HLA-A^∗^68:02, HLA-A^∗^02:17	80.95%	Yes	Yes
NLPHVVPRY	25–33	0.6878	HLA-B^∗^27:20, HLA-A^∗^32:07, HLA-A^∗^68:23, HLA-C^∗^12:03, HLA-A^∗^02:17, HLA-B^∗^40:13, HLA-C^∗^14:02, HLA-C^∗^07:02, HLA-A^∗^32:15, HLA-B^∗^15:02	80.95%	Yes	Yes
NTIPAISGL	54–62	0.7847	HLA-A^∗^68:23, HLA-C^∗^03:03, HLA-A^∗^68:02, HLA-A^∗^02:50, HLA-A^∗^32:07, HLA-B^∗^15:02, HLA-B^∗^27:20, HLA-A^∗^26:02, HLA-C^∗^12:03, HLA-A^∗^02:17, HLA-A^∗^02:06, HLA-A^∗^69:01, HLA-C^∗^07:02, HLA-A^∗^32:15, HLA-B^∗^15:17	80.95%	Yes	Yes
VPRYHSIDV	30–38	1.5628	HLA-A^∗^68:23, HLA-B^∗^27:20, HLA-C^∗^12:03, HLA-A^∗^32:07, HLA-B^∗^07:02, HLA-B^∗^42:01	80.95%	No	Yes
IPAISGLGV	56–64	1.2054	HLA-A^∗^02:50, HLA-B^∗^27:20, HLA-A^∗^32:07, HLA-A^∗^68:23, HLA-C^∗^12:03, HLA-B^∗^42:01, HLA-B^∗^07:02, HLA-B^∗^40:13, HLA-A^∗^32:15	76.19%	Yes	Yes
YLKNHNIDL	26–34	1.7400	HLA-C^∗^12:03, HLA-B^∗^15:02, HLA-A^∗^02:50, HLA-A^∗^68:23, HLA-A^∗^32:07, HLA-C^∗^14:02, HLA-C^∗^03:03, HLA-B^∗^27:20, HLA-A^∗^02:12, HLA-A^∗^02:17, HLA-A^∗^02:03, HLA-C^∗^06:02, HLA-A^∗^32:15, HLA-B^∗^08:01, HLA-A^∗^02:02, HLA-C^∗^07:02	71.43%	Yes	Yes
YNVAWRTYL	19–27	0.4500	HLA-C^∗^03:03, HLA-A^∗^02:50, HLA-B^∗^27:20, HLA-B^∗^15:02, HLA-A^∗^68:23, HLA-A^∗^32:15, HLA-C^∗^12:03, HLA-C^∗^14:02, HLA-A^∗^32:07, HLA-A^∗^02:17	71.43%	No	Yes
LEPIIGDKL	54–62	0.4388	HLA-A^∗^02:17, HLA-A^∗^02:50, HLA-C^∗^12:03, HLA-A^∗^32:07, HLA-A^∗^68:23, HLA-B^∗^40:13, HLA-B^∗^27:20, HLA-B^∗^40:01, HLA-A^∗^32:15, HLA-B^∗^15:02	80.95%	No	Yes
CEEYGCLAI	1048–1056	1.6829	HLA-B^∗^27:20, HLA-C^∗^12:03, HLA-A^∗^32:07, HLA-B^∗^40:13, HLA-B^∗^40:01, HLA-A^∗^02:50, HLA-A^∗^68:23, HLA-C^∗^05:01, HLA-A^∗^32:15, HLA-C^∗^03:03	95.24%	Yes	Toxin
NSGPYNVAW	15–23	0.8498	HLA-B^∗^27:20, HLA-A^∗^32:07, HLA-C^∗^12:03, HLA-B^∗^40:13, HLA-A^∗^32:15, HLA-A^∗^68:23, HLA-C^∗^07:01, HLA-C^∗^03:03, HLA-B^∗^58:01, HLA-A^∗^02:50, HLA-B^∗^57:01	80.95%	No	Yes
RQHRGCIRF	43–51	1.0559	HLA-B^∗^27:20, HLA-B^∗^15:03, HLA-A^∗^32:07, HLA-B^∗^40:13, HLA-A^∗^68:23, HLA-B^∗^15:01, HLA-C^∗^12:03	71.43%	Yes	Toxin
AISGLGVGY	58–66	1.1548	HLA-A^∗^32:07, HLA-C^∗^12:03, HLA-A^∗^32:15, HLA-C^∗^03:03, HLA-A^∗^68:23, HLA-B^∗^27:20, HLA-B^∗^40:13, HLA-A^∗^30:02, HLA-B^∗^15:17	76.19%	No	Yes

**Table 2:** MHC-II alleles for the desired epitope.

Core sequence	MHC-II alleles	position	Peptide	IC50 value
TSSWGCEEY	HLA-DQA1^∗^01:01/DQB1^∗^05:01	1040–1054	KEHTSSWGCEEYGCL	2715
	HLA-DQA1^∗^01:01/DQB1^∗^05:01	1041–1055	EHTSSWGCEEYGCLA	2801

LAIDTGCLY	HLA-DRB3^∗^01:01	1049–1063	EEYGCLAIDTGCLYG	194
	HLA-DRB3^∗^01:01	1050–1064	EYGCLAIDTGCLYGS	195
	HLA-DRB3^∗^01:01	1052–1066	GCLAIDTGCLYGSCQ	195
	HLA-DRB3^∗^01:01	1051–1065	YGCLAIDTGCLYGSC	196
	HLA-DRB1^∗^03:01	1049–1063	EEYGCLAIDTGCLYG	401
	HLA-DRB1^∗^03:01	1050–1064	EYGCLAIDTGCLYGS	401
	HLA-DRB1^∗^03:01	1051–1065	YGCLAIDTGCLYGSC	403
	HLA-DRB1^∗^03:01	1052–1066	GCLAIDTGCLYGSCQ	405
	HLA-DRB3^∗^01:01	1054–1068	LAIDTGCLYGSCQDV	601
	HLA-DRB3^∗^01:01	1053–1067	CLAIDTGCLYGSCQD	611
	HLA-DRB1^∗^03:01	1054–1068	LAIDTGCLYGSCQDV	1173
	HLA-DRB1^∗^03:01	1053–1067	CLAIDTGCLYGSCQD	1200
	HLA-DRB1^∗^12:01	1051–1065	YGCLAIDTGCLYGSC	1450
	HLA-DRB1^∗^12:01	1050–1064	EYGCLAIDTGCLYGS	1457
	HLA-DPA1^∗^01:03/DPB1^∗^02:01	1049–1063	EEYGCLAIDTGCLYG	1545
	HLA-DPA1^∗^01:03/DPB1^∗^02:01	1051–1065	YGCLAIDTGCLYGSC	1551
	HLA-DPA1^∗^01:03/DPB1^∗^02:01	1050–1064	EYGCLAIDTGCLYGS	1554
	HLA-DRB1^∗^12:01	1052–1066	GCLAIDTGCLYGSCQ	1561
	HLA-DPA1^∗^01:03/DPB1^∗^02:01	1052–1066	GCLAIDTGCLYGSCQ	1571
	HLA-DRB1^∗^01:01	1052–1066	GCLAIDTGCLYGSCQ	1597
	HLA-DPA1^∗^02:01/DPB1^∗^01:01	1050–1064	EYGCLAIDTGCLYGS	2005
	HLA-DRB1^∗^13:02	1050–1064	EYGCLAIDTGCLYGS	2117
	HLA-DPA1^∗^02:01/DPB1^∗^01:01	1051–1065	YGCLAIDTGCLYGSC	2123
	HLA-DRB1^∗^13:02	1049–1063	EEYGCLAIDTGCLYG	2127
	HLA-DRB1^∗^13:02	1051–1065	YGCLAIDTGCLYGSC	2145
	HLA-DPA1^∗^02:01/DPB1^∗^01:01	1052–1066	GCLAIDTGCLYGSCQ	2201
	HLA-DPA1^∗^02:01/DPB1^∗^01:01	1049–1063	EEYGCLAIDTGCLYG	2287
	HLA-DRB1^∗^04:01	1050–1064	EYGCLAIDTGCLYGS	2673
	HLA-DRB1^∗^12:01	1053–1067	CLAIDTGCLYGSCQD	2760
	HLA-DRB1^∗^12:01	1054–1068	LAIDTGCLYGSCQDV	2917
	HLA-DRB1^∗^12:01	1049–1063	EEYGCLAIDTGCLYG	2949

**Table 3:** Linear B-cell epitopes of the polyprotein of OROV predicted by BepiPred-2.0.

B-cell epitopes	Position	Antigenicity score	Conservancy (%)	Toxicity	Allergenicity	
					AllerTOP 2.0	AllergenFP 1.0
HPLSTSQIGDRC	17–28	1.2943	94.44	No	No	No
FKEMNLSVGL	35–44	1.9084	100.00	No	Yes	Yes
FSKNTLALEA	62–71	0.4645	5.56	No	Yes	No
VKDWSECNPVLDKL	81–94	−0.0129	5.56	No	No	No
SCTEALKVHRMGKDCLGYKS	282–301	0.4510	100.00	No	Yes	No
AGERMYKLEELADD	334–347	0.2927	38.89	No	Yes	No
IHYRPGLKVDLTK	404–416	0.4322	33.33	No	Yes	No
FDEQQSSGFEYEVFLKDMHIQRESCKFGPR	427–456	0.6740	5.56	No	Yes	Yes
DCLSKNIKITYQELH	484–498	1.2448	11.11	No	Yes	No
YIMGDTCVSKSELYSDLLSKNLITEYDKKYFEPDTVN	504–540	0.1143	5.56	No	No	No
ECEMLSLKKNSGPYN	566–580	0.6930	100.00	No	Yes	No
LCSRHNY	594–600	1.2777	100.00	No	Yes	Yes
HSMCKNTDIDFNNEIETYYKANAAAY	610–635	0.7190	5.56	No	Yes	No
NISLGRSTRSAQHSIMTNEIPKSNPFTDYSYSSVNIKECMSP	699–740	0.6399	5.56	No	Yes	Yes
KKRDSVPHT	747–755	0.9721	11.11	No	No	Yes
KYKAFDWPEIETVQKGQKLCL	765–785	1.3196	33.33	No	Yes	No
SHCNLEFTAITADKIMSL	788–805	1.0518	83.33	No	No	No
NCYKESFTAQPADMQTGI	807–824	0.8661	61.11	No	No	No
CITLEDKA	835–842	2.5070	5.56	No	No	Yes
KYYYSDGKEHAKDGSIN	851–867	0.6483	33.33	No	No	No
CSEQRFPIHKNWFKKCNWDKTHKEFTTMRQINYNDITSYRKAIES	875–919	0.1740	27.78	No	No	No
GTDLMTHHYKPTKNLPHVVPRYH	922–944	0.3102	72.22	No	No	No
AQYQKVYTTGPSISINIEHNEK	999–1020	0.6311	33.33	No	Yes	No
PEKIPAKEGWLTFSKEHTSSW	1026–1046	0.5512	100.00	No	No	No
ETYCNDMDILEPIIGD	1095–1110	0.1167	100.00	No	No	Yes
TQTNQLP	1119–1125	0.9980	100.00	No	Yes	Yes
TGQINDVGNTALQCGSIQVVNGSTIGSGNPK	1137–1167	0.8145	33.33	No	Yes	Yes
FNDNYQSCT	1186–1194	0.0858	100.00	No	No	Yes
DARNDLIPYRKG	1197–1208	1.4056	5.56	No	No	No
DINYKIFTKSV	1231–1241	0.3093	55.56	No	No	No
TVKPYNQKIDLSQLDESNYIKEEDLQCG	1322–1349	0.8134	38.89	No	No	No

**Table 4:** Linear B-cell epitopes of the polyprotein of OROV predicted by LBtope.

B-cell epitopes	Position	Antigenicity scores	Conservancy (%)	Toxicity	Allergenicity	
					AllerTOP 2.0	AllergenFP 1.0
IAYGHPLSTSQI	13–24	0.3474	50.00	No	Yes	No
TTAFSKNTLALEAT	59–72	0.6595	5.56	No	Yes	No
PRMYTCRAACDI	110–121	−0.5422	5.56	No	Yes	No
GLKVDLTKTN	409–418	0.5124	33.33	No	Yes	Yes
SSGFEYEVFLKDMH	432–445	0.1548	38.89	No	No	No
QRESCKFGPRVNHFRNTK	447–464	1.1279	38.89	No	No	No
DLLSKNLITEYDKKYFEPDTVNDQFNKIEFAQD	519–551	0.2055	33.33	No	No	No
DFNNEIETYYKANAAAYRSDFYTI	619–642	0.1701	5.56	No	Yes	No
TRSAQHSIMTN	706–716	0.1229	38.89	No	Yes	No
HTNHLLCKMDNKYKAFDWPEIETVQK	754–779	1.0858	16.67	No	Yes	No
CITLEDKAWP	835–844	2.0270	5.56	No	No	Yes
CGDKYYYSDGKEHAKDGS	848–865	0.3358	33.33	No	Yes	No
KCNWDKT	889–895	0.4951	100.00	Yes	Yes	No
HHYKPTKNLPHVVPRYH	928–944	0.6022	100.00	No	No	No
VYTTGPSIS	1004–1013	0.4965	38.89	No	Yes	Yes
CITLPHETYCNDMDILEPII	1089–1108	0.2420	100.00	No	Yes	Yes
DNYQSCTRLDARNDLIPYRKG	1188–1208	1.0163	5.56	No	Yes	No
CHCKTNCED	1273–1281	1.1907	100.00	Yes	No	Yes
NQKIDLSQL	1327–1335	0.5667	100.00	No	Yes	Yes

**Table 5:** Most conserved B-cell epitope sequences predicted by both BepiPred-2.0 and LBtope.

B-cell epitopes	Position	Length	Antigenicity score	Conservancy (%)	Toxicity	Allergenicity	
						AllerTOP 2.0	AllergenFP 1.0
HPLSTSQI	17–24	8	0.7973	94.44	No	Yes	Yes
FSKNTLALEA	62–71	10	0.4645	5.56	No	Yes	No
GLKVDLTK	409–416	8	−0.8608	33.33	No	Yes	Yes
SSGFEYEVFLKDMH	432–445	14	0.1548	38.89	No	No	No
QRESCKFGPR	447–456	10	2.2285	38.89	No	No	Yes
DLLSKNLITEYDKKYFEPDTVN	519–540	22	0.2587	33.33	No	No	No
DFNNEIETYYKANAAAY	619–635	17	0.1778	5.56	No	No	No
TRSAQHSIMTN	706–716	11	0.1229	38.89	No	Yes	No
KYKAFDWPEIETVQK	765–779	15	1.1837	33.33	No	Yes	No
CITLEDKA	835–842	8	2.5070	5.56	No	No	Yes
KYYYSDGKEHAKDGS	851–865	15	0.5485	33.33	No	No	No
KCNWDKT	889–895	7	0.4951	100.00	Yes	Yes	No
HHYKPTKNLPHVVPRYH	928–944	17	0.6022	100.00	No	No	No
VYTTGPSIS	1004–1013	9	0.4965	38.89	No	Yes	Yes
ETYCNDMDILEPII	1095–1108	14	0.1553	100.00	No	No	No
DNYQSCT	1188–1194	7	−0.1560	100.00	No	No	Yes
DARNDLIPYRKG	1197–1208	12	1.4056	5.56	No	No	No
NQKIDLSQL	1327–1335	9	0.5667	100.00	No	Yes	Yes

**Table 6:** Predicted conformational B-cell epitopes of the polyprotein of OROV.

Epitope no.	Residues and their positions	No. of residues	Score	3D structure
1.	V992, K993, K994, A995, V996, Y997, K998, A999, Q1000, Y1001, Q1002, K1003, V1004, T1006, T1007, G1008, P1009, S1010, I1011, S1012, I1013, N1014, I1015, E1016, H1017, N1018, E1019, K1020, C1021, T1022, G1023, H1024, C1025, P1026, E1027, K1028, I1029, P1030, A1031, K1032, E1033, G1034, W1035, L1036, T1037, K1040, E1041, S1044, S1045, W1046, G1047, C1048, E1049, E1050, Y1051, G1052, C1053, L1054, A1055, I1056, D1057, T1058, G1059, C1060, L1061, Y1062, G1063, S1064, C1065, Q1066, D1067, V1068, I1069, R1070, P1071, E1072, L1073, D1074, I1075, K1077, K1078, I1079, G1080, S1081, E1082, D1110, K1111, I1140	88	0.906	Figure 5(a)
2.	Y76, R77, I80, V81, D83, W84, S85, E86, C87, N88, P89, V90, L91, D92, K93, L94, G95, N96, M98, N105, G106, H107, L108, V109, P110, R111, M112, Y113, T114, C115, R116, A117, A118, C119, D120, I121, R122, L123, N124, K125, D126, N127, A128, E129, I130, I131, L132, S133, S134, T135, K136, L137, N138, H139, F140, E141, I142, V143, G144, T145, T146, S147, T148, S149, G150, W151, F152, K153, N154	69	0.895	Figure 5(b)
3.	M738, S739, P740, S742, C760, K761, M762, D763, N764, K765, Y766, K767, A768, F769, D770, W771, P772, E773, I774, E775, T776, V777, Q778, K779, G780, Q781, K782, L783, C784, L785, G786, D787, S788, H789, C790, K810, E811, S812, F813, T814, A815, Q816, P817	43	0.875	Figure 5(c)
4.	Y563, T565, E566, C567, E568, M569, L570, S571, L572, K573, K574, N575, S576, G577, P578	15	0.864	Figure 5(d)
5.	G43, L44, G45, E46, I47, C48, V49, K50, D51, D52, I53, S54, I55	13	0.845	Figure 5(e)
6.	I1385, I1388, V1389, L1390, V1394, L1395, L1396, P1397, I1398, G1399, R1400, L1401, L1402, K1403, A1404, F1405, L1406, I1407, K1408, E1412	20	0.843	Figure 5(f)
7.	M292, D295, C296, L297, G298	5	0.838	Figure 5(g)
8.	P332, I333, A334, G335, E336, R337, M338, Y339, K340, E342, E343, A345, D346, F387, N388, Y389, L390, S397, C398, S399, M400, C401, G402, L403, I404, H405, Y406, R407, P408, G409, L410, K411, V412, D413, L414, T415, K416, T417, N418, K419, C420, G421, S422	43	0.837	Figure 5(h)

**Table 7:** Different physicochemical properties of the Oropouche virus (OROV) polyprotein.

Features	Value
Number of amino acids	1420
Molecular weight	161275.63
Theoretical pI	8.07
Total number of negatively charged residues (Asp + Glu)	151
Total number of positively charged residues (Arg + Lys)	161
Total number of atoms	22,605
Extinction coefficients	177,070
Instability index	37.22
Aliphatic index	88.99
Grand average of hydropathicity (GRAVY)	−0.138

**Table 8:** Top 10 binding pockets with binding residues, energy, and volume of OROV polyprotein.

Rank	Energy (kcal/mol)	Energy Range	Volume (Å)	Certer X	Certer Y	Certer Z	Residues
1	−1677.27	(−14.79, −8.90)	156.00	123.724	99.245	115.842	Gly-323, Leu-324, Leu-326, Gln-430, Gln-431, Ser-432, Ser-433, Gly-434, Phe-435, Glu-436, Phe-453, Lys-647, Thr-648, Ala-649, Phe-650, Cys-835, Ile-836, Leu-838
2	−1677.27	(−14.79, −8.90)	156.00	123.724	99.245	115.842	Asp-750, Ser-751, Val-752, Ser-828, Asp-830, Ile-832, Gly-833, Ile-836, Thr-837, Cys-870, Leu-871, Ala-872, Asn-873, Lys-874, Glu-877, Phe-880, Pro-881, His-928, His-929, Tyr-930, Lys-931, Pro-932
3	−1677.27	(−14.79, −8.90)	156.00	123.724	99.245	115.842	Lys-744, Cys-745, Phe-746, Lys-747, Lys-748, Leu-758, Leu-759, Cys-760, Thr-776, Gln-778, Lys-779, Gly-780, Gln-781, Lys-782, Leu-783, His-789, Phe-813
4	−1677.27	(−14.79, −8.90)	156.00	123.724	99.245	115.842	Leu-34, Phe-35, Lys-36, Glu-37, Asn-39, Phe-178, His-179, Cys-181, Ala-264, Ser-265, Pro-267, Phe-268, Phe-281, Cys-309, Ser-311, Lys-312, Trp-314
5	−1677.27	(−14.79, −8.90)	156.00	123.724	99.245	115.842	Phe-79, Asp-83, Glu-86, Met-98, Val-99, Leu-100, Asn-101, Val-102, Asp-103, Asn-105, Gly-106, Val-109, Tyr-113, Arg-116, Asp-120, Asp-126, Asn-127, Ala-128, Glu-129, Ile-131, Leu-132, Asn-138, Ile-142
6	−1677.27	(−14.79, −8.90)	156.00	123.724	99.245	115.842	Ile-472, Ser-475, Phe-476, Val-581, Arg-584, Thr-585, Leu-587, Lys-588, Ala-630, Ala-632, Ala-633, Arg-636, Ser-637, Asp-638, Phe-639, Tyr-640, Thr-641
7	−1677.27	(−14.79, −8.90)	156.00	123.724	99.245	115.842	Ser-912, Tyr-913, Ile-958, Asn-959, Gly-960, Phe-961, Ile-962, Gln-963, Cys-1301, Lys-1302, Ser-1303, Lys-1304, Lys-1324, Leu-1332, Ser-1333, Gln-1334, Phe-1364, Lys-1365, Gly-1366
8	−1677.27	(−14.79, −8.90)	156.00	123.724	99.245	115.842	Ile-962, Gln-963, Asn-964, Thr-965, Ile-966, Lys-1304, Asn-1311, Gln-1320, Leu-1321, Thr-1322, Lys-1324, Leu-1367, Phe-1368, Ser-1369, Tyr-1413
9	−1677.27	(−14.79, −8.90)	156.00	123.724	99.245	115.842	Tyr-661, Val-690, Leu-691, Lys-692, Phe-693, Ala-694, Leu-697, Leu-698, Ser-701, Arg-914, Lys-915, Glu-918, Ser-919, Gly-922, Thr-923
10	−1677.27	(−14.79, −8.90)	156.00	123.724	99.245	115.842	Ile-255, Asn-256, Ser-257, Ser-259, Asn-260, Cys-261, Leu-262, Phe-268, Cys-271, Pro-272, Cys-283, Thr-284, Ala-286, Leu-287, His-290, Lys-310, Ser-311, Lys-312

## Data Availability

The data used to support the findings of this study are available from the corresponding author upon request.

## Abbreviations

OROV: Oropouche virus
CNS: Central nervous system
CTL: Cytotoxic T-lymphocytes
FAO: Food and Agriculture Organization
GRAVY: Grand average hydropathicity
HMM: Hidden Markov model
IRF-3: Interferon regulatory transcription factor
MAVS: Mitochondrial antiviral signaling protein
MHC-I: Major histocompatibility complex class I
MHC-II: Major histocompatibility complex class II
PI: Protrusion index
PMDB: Protein model database
PDB: Protein Data Bank
SMM: Stabilized matrix base method
SVM: Support vector machine
TAP: Transport-associated protein
WHO: World Health Organization.

## Supplementary Materials

Supplementary 1 Figure S1: docking simulation study (control). (A) The docking results as the surface structure of the MHC-I HLA allele “HLA-B^∗^35:01” (cyan color) and the sticky form of epitope “LPFERATVM” (red color). The black color box represents the position of epitope in the surface structure. (B) Epitope in the cartoon structure. (C) Sticky form of epitope “LPFERATVM” (red color) with residue position. (D) Binding interaction of the epitope residues and the HLA-B^∗^35:01 residues through the hydrogen bonds. Hydrogen bonds and the distances are shown in green color.

Supplementary 2 Figure S2: secondary structure of the OROV polyprotein, ALB07207.

Supplementary 3 Figure S3: Ramachandran plot analysis of OROV polyprotein. Here, the red region indicates the favored region, the yellow region for the allowed region, light yellow for the generously allowed region, and white for the disallowed region. Phi and Psi angles determine torsion angles.

Supplementary 4 Figure S4: ProSA—web results of the OROV polyprotein. (A) ProSA—web *z*-scores of all protein chains in PDB determined by X-ray crystallography (light blue) or NMR spectroscopy (dark blue) with respect to their length. The *z*-score of the OROV polyprotein is highlighted as large dots. (B) Energy plot of the OROV polyprotein. Residue energies averaged over a sliding window are plotted as a function of the central residue in the window. (C) Jmol C*^α^* trace of the OROV polyprotein. Residues are colored from blue to red in the order of increasing residue energy.

Supplementary 5 Figure S5: QMEAN score and QMEANDisCo score of the OROV polyprotein. (A) QMEAN4 score with linear combination of four statistical potential terms. (B) QMEAN6 score with two additional agreement terms evaluating the consistency of structural features with sequence-based predictions. (C) Predicted local similarity to target structure. (D) QMEANDisCo scoring plot. It compares local QMEAN scores with local DisCo scores and the resulting QMEANDisCo scores. Depending on the situation of finding homologous, DisCo is not necessarily defined everywhere.

Supplementary 6 Figure S6: hydrophobicity plot analysis of OROV polyprotein, ALB07207.

Supplementary 7 Table S1: total 128 epitopes predicted for 12 super types. (√) indicating the acceptance of the epitope which has both positive antigenicity and immunogenicity scores.

Supplementary 8 Table S2: 37 CD8^+^ T-cell epitopes and their combined score, antigenicity, immunogenicity, conservancy, and MHC-I interaction.

Supplementary 9 Table S3: population coverage for the selected 18 epitopes and their HLA alleles.
