# Supplementary figures and images for: Immunoinformatics Approach for Epitope-Based Peptide Vaccine Design and Active Site Prediction against Polyprotein of Emerging Oropouche Virus
Source: J Immunol Res. 2018 Oct 8;2018:6718083. doi: 10.1155/2018/6718083 (PMC6196980; doi:10.1155/2018/6718083)

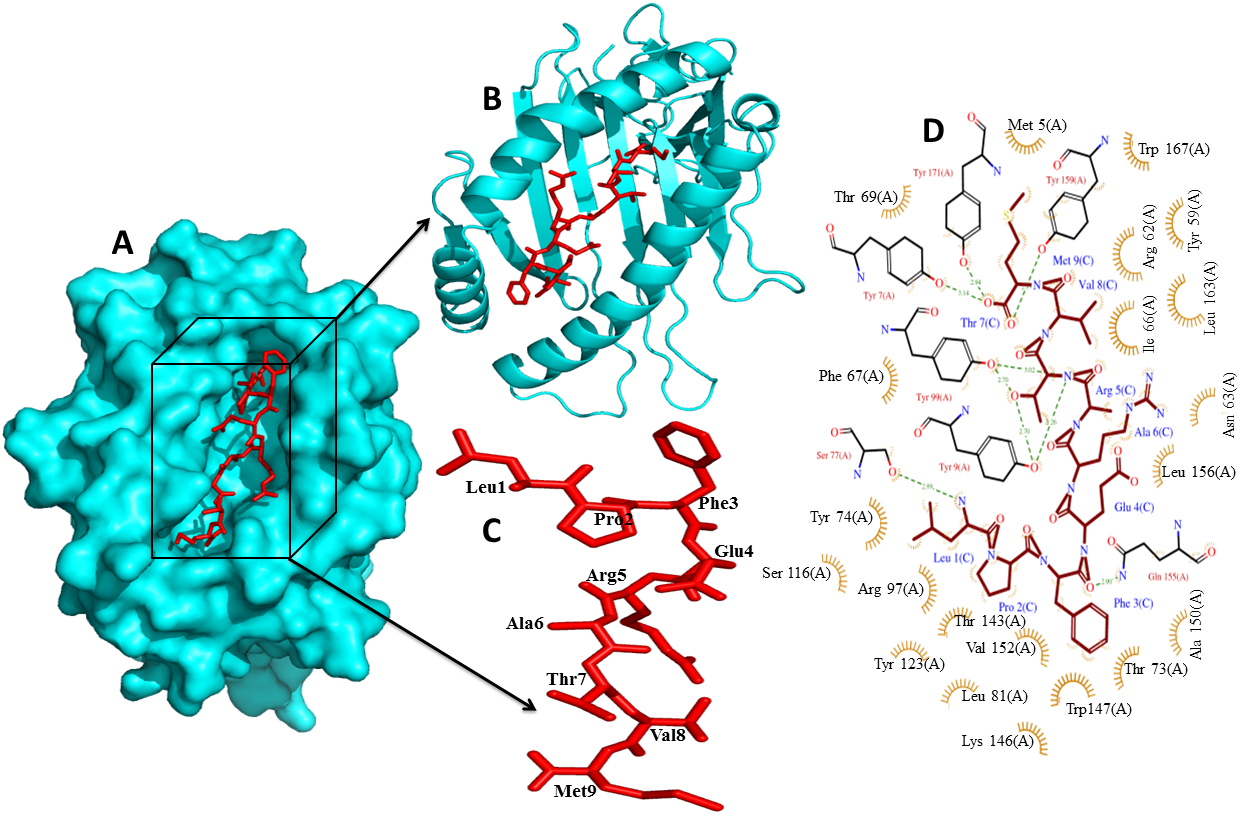

Supplement: Supplementary 1 — Figure S1: docking simulation study (control). (A) The docking results as the surface structure of the MHC-I HLA allele “HLA-B∗35:01” (cyan color) and the sticky form of epitope “LPFERATVM” (red color). The black color box represents the position of epitope in the surface structure. (B) Epitope in the cartoon structure. (C) Sticky form of epitope “LPFERATVM” (red color) with residue position. (D) Binding interaction of the epitope residues and the HLA-B∗35:01 residues through the hydrogen bonds. Hydrogen bonds and the distances are shown in green color. [file 6718083.f1.docx]

**
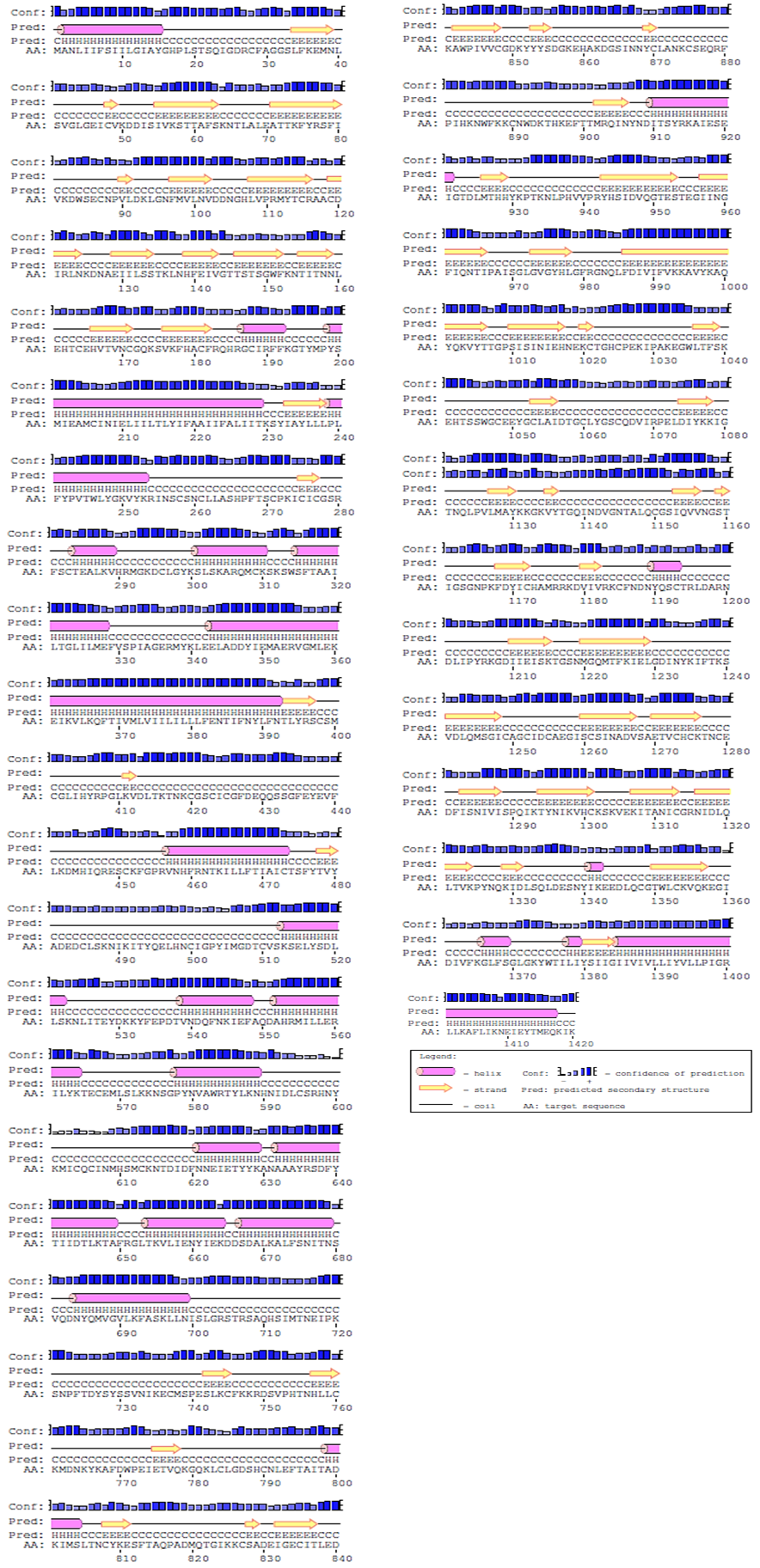
**

Supplement: Supplementary 2 — Figure S2: secondary structure of the OROV polyprotein, ALB07207. [file 6718083.f2.docx]

**
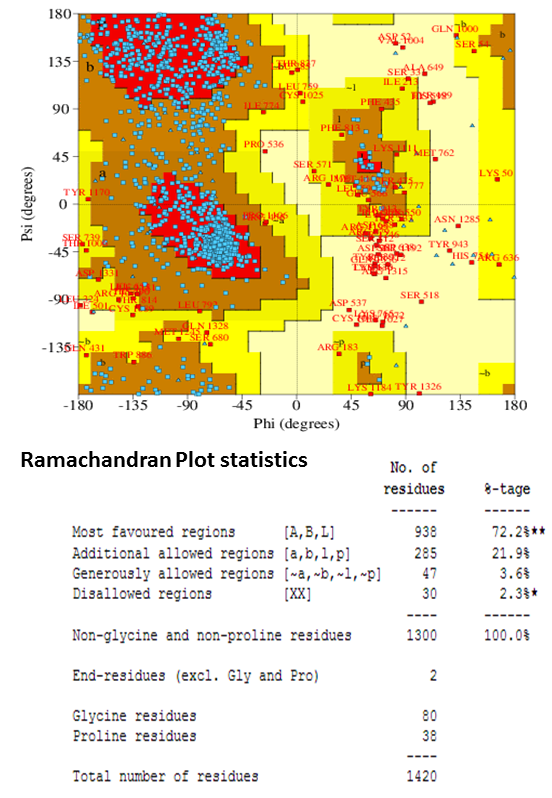
**

Supplement: Supplementary 3 — Figure S3: Ramachandran plot analysis of OROV polyprotein. Here, the red region indicates the favored region, the yellow region for the allowed region, light yellow for the generously allowed region, and white for the disallowed region. Phi and Psi angles determine torsion angles. [file 6718083.f3.docx]

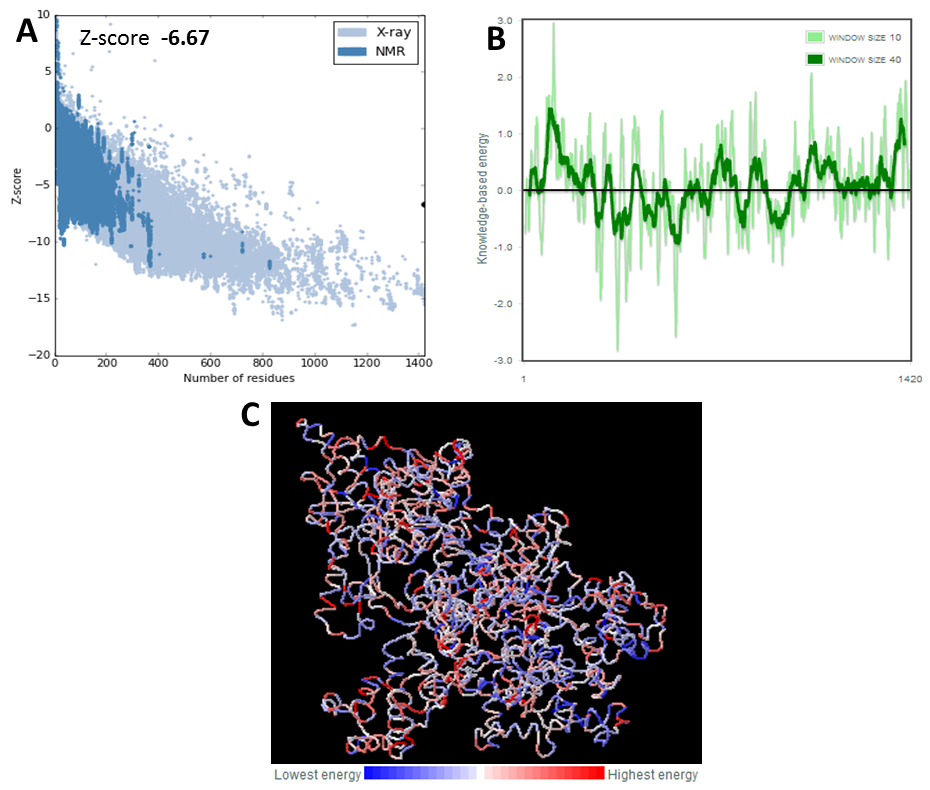

Supplement: Supplementary 4 — Figure S4: ProSA—web results of the OROV polyprotein. (A) ProSA—web z-scores of all protein chains in PDB determined by X-ray crystallography (light blue) or NMR spectroscopy (dark blue) with respect to their length. The z-score of the OROV polyprotein is highlighted as large dots. (B) Energy plot of the OROV polyprotein. Residue energies averaged over a sliding window are plotted as a function of the central residue in the window. (C) Jmol Cα trace of the OROV polyprotein. Residues are colored from blue to red in the order of increasing residue energy. [file 6718083.f4.docx]

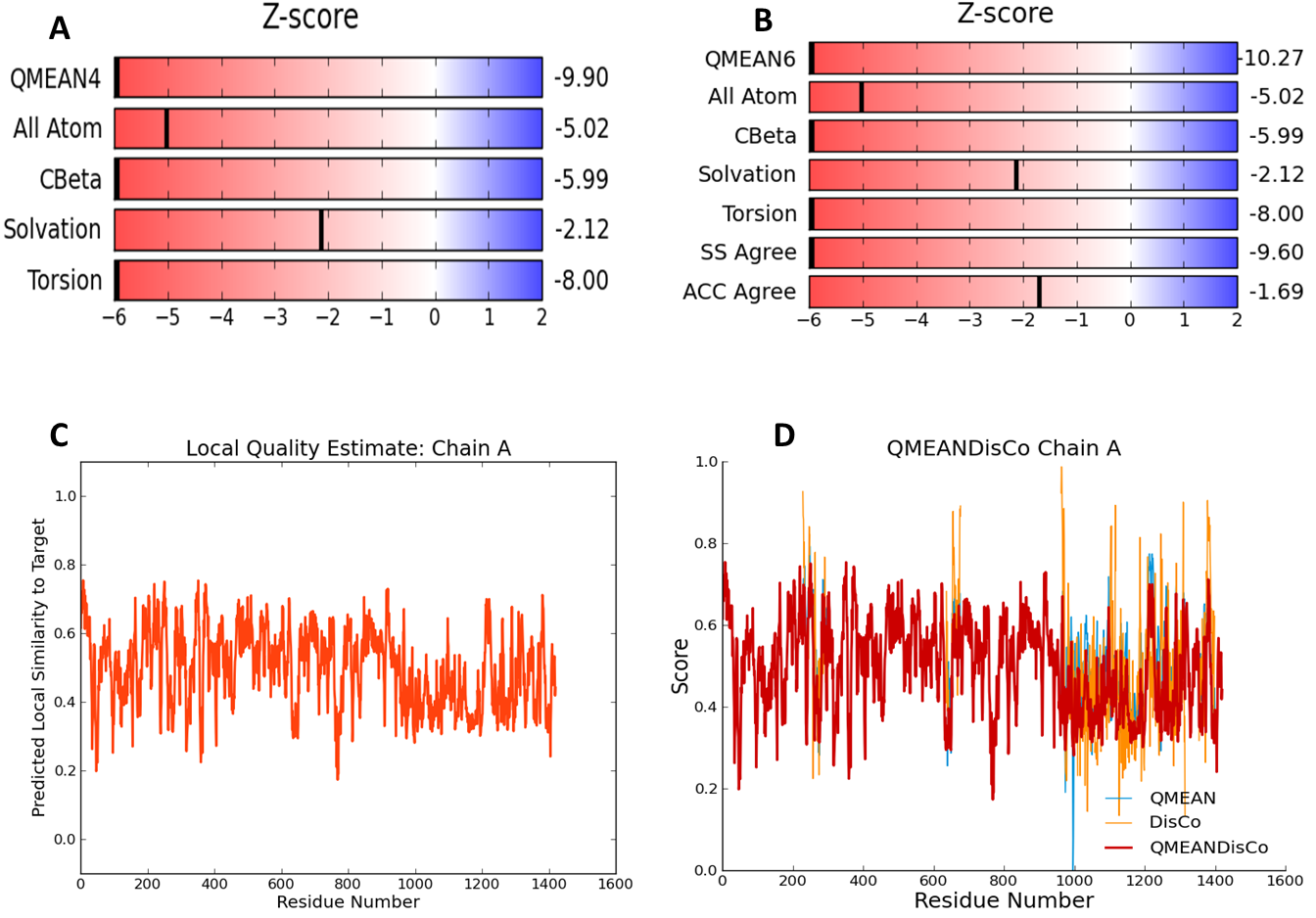

Supplement: Supplementary 5 — Figure S5: QMEAN score and QMEANDisCo score of the OROV polyprotein. (A) QMEAN4 score with linear combination of four statistical potential terms. (B) QMEAN6 score with two additional agreement terms evaluating the consistency of structural features with sequence-based predictions. (C) Predicted local similarity to target structure. (D) QMEANDisCo scoring plot. It compares local QMEAN scores with local DisCo scores and the resulting QMEANDisCo scores. Depending on the situation of finding homologous, DisCo is not necessarily defined everywhere. [file 6718083.f5.docx]

**
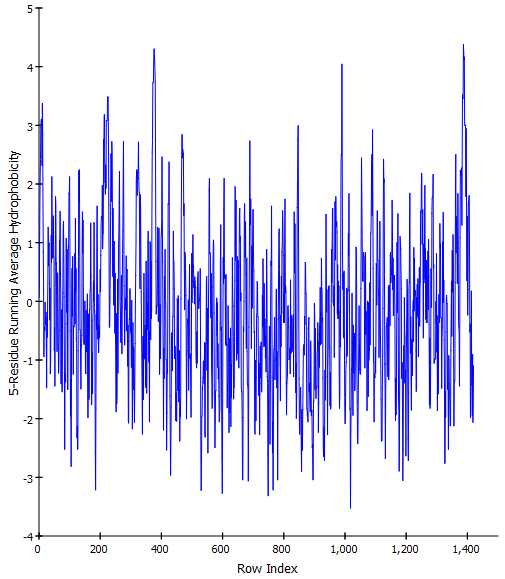
**

Supplement: Supplementary 6 — Figure S6: hydrophobicity plot analysis of OROV polyprotein, ALB07207. [file 6718083.f6.docx]
