# Supplementary material for: Immunoinformatics Approach for Epitope-Based Peptide Vaccine Design and Active Site Prediction against Polyprotein of Emerging Oropouche Virus
Source: J Immunol Res. 2018 Oct 8;2018:6718083. doi: 10.1155/2018/6718083 (PMC6196980; doi:10.1155/2018/6718083)
Supplement: Supplementary 7 — Table S1: total 128 epitopes predicted for 12 super types. (√) indicating the acceptance of the epitope which has both positive antigenicity and immunogenicity scores. [file 6718083.f7.docx]

**Table S1:** Total 128 epitopes predicted for 12 super types. (√) indicating the acceptance of epitope which has both positive antigenicity and immunogenicity score.

| No. | Epitopes | Combined score | Vaxijen score | Immunogenicity | Remarks |
| --- | --- | --- | --- | --- | --- |
|  | GTDLMTHHY | 3.6869 | -0.1743 | -0.09222 | × |
|  | FTTMRQINY | 2.686 | 1.2368 | -0.1142 | × |
|  | FTDYSYSSV | 2.41 | 1.4972 | -0.39738 | × |
|  | ITNSVQDNY | 2.3631 | 0.5318 | -0.22247 | × |
|  | TSSWGCEEY | 2.1681 | 0.9867 | 0.29444 | √ |
|  | TIAICTSFY | 2.1583 | -0.2163 | 0.05944 | × |
|  | FSIILGIAY | 2.1233 | 1.0143 | 0.3334 | √ |
|  | LSKNLITEY | 2.0798 | -0.6870 | 0.12923 | × |
|  | CSMCGLIHY | 1.9016 | 0.5400 | 0.04253 | √ |
|  | LTKVLIENY | 1.8861 | -0.3305 | 0.16674 | × |
|  | AAAYRSDFY | 1.7324 | 0.5498 | -0.00923 | × |
|  | LSKNIKITY | 1.5811 | 1.7710 | -0.01491 | × |
|  | ALEATTKFY | 1.5358 | 0.6372 | 0.03261 | √ |
|  | TTSTSGWFK | 1.5285 | -0.3627 | 0.1115 | × |
|  | KLEELADDY | 1.5185 | -0.1743 | 0.19096 | × |
|  | DSDALKALF | 1.4779 | -0.4752 | -0.14069 | × |
|  | LLCKMDNKY | 1.463 | 1.2590 | -0.51608 | × |
|  | LSQLDESNY | 1.3946 | 0.4738 | -0.07631 | × |
|  | LAIDTGCLY | 1.5886 | 0.9551 | 0.08324 | √ |
|  | QLFDIVIFV | 1.4923 | 0.2479 | 0.4095 | × |
|  | ILMEFVSPI | 1.4699 | -0.2148 | 0.05051 | × |
|  | LLLPLFYPV | 1.4238 | 0.4221 | 0.07504 | √ |
|  | SLIEICITL | 1.4066 | 0.9867 | 0.3578 | √ |
|  | IIFSIILGI | 1.3102 | 0.5287 | 0.13685 | √ |
|  | YIAYLLLPL | 1.2739 | 0.5558 | -0.0281 | × |
|  | VMLVIILIL | 1.2554 | 0.5008 | 0.36122 | √ |
|  | ILLERILYK | 1.718 | -0.7354 | 0.26131 | × |
|  | IIFALIITK | 1.6291 | 0.7267 | 0.32685 | √ |
|  | RLLKAFLIK | 1.6251 | -1.1711 | -0.0039 | × |
|  | KFYRSFIVK | 1.5817 | -1.2421 | 0.13642 | × |
|  | KTAFRGLTK | 1.5451 | -0.3129 | 0.22612 | × |
|  | MTHHYKPTK | 1.4987 | 0.4698 | -0.15023 | × |
|  | CLGYKSLSK | 1.4629 | 1.0317 | -0.46447 | × |
|  | KLLNISLGR | 1.4333 | 1.0703 | -0.0258 | × |
|  | CINMHSMCK | 1.4293 | 0.5308 | -0.48273 | × |
|  | ELYSDLLSK | 1.4196 | -0.3028 | -0.26253 | × |
|  | LIHYRPGLK | 1.4059 | 1.7718 | 0.06886 | √ |
|  | AVYKAQYQK | 1.3963 | -0.4888 | -0.35994 | × |
|  | NVAWRTYLK | 1.3838 | 0.2563 | 0.31293 | × |
|  | KIMSLTNCY | 1.3781 | 0.1715 | -0.23469 | × |
|  | RVNHFRNTK | 1.3414 | 0.4608 | 0.21039 | √ |
|  | TLALEATTK | 1.3029 | 1.3929 | 0.19131 | √ |
|  | LSKARQMCK | 1.2934 | 1.2171 | -0.26897 | × |
|  | LLPIGRLLK | 1.2906 | 0.1025 | 0.1962 | × |
|  | HLLCKMDNK | 1.2746 | 1.6913 | -0.41821 | × |
|  | IMSLTNCYK | 1.272 | 0.4921 | -0.08081 | × |
|  | CFAGGSLFK | 1.2605 | 0.0517 | -0.01689 | × |
|  | LYIFAAIIF | 1.7417 | 0.2339 | 0.42601 | × |
|  | AYRSDFYTI | 1.7325 | 0.1943 | 0.00169 | × |
|  | DYSYSSVNI | 1.6369 | 1.3354 | -0.34319 | × |
|  | TYQELHNCI | 1.6251 | -0.1348 | 0.04584 | × |
|  | KFGPRVNHF | 1.6175 | 1.4607 | 0.10254 | √ |
|  | LYGKVYKRI | 1.6029 | 0.0616 | -0.32104 | × |
|  | RFPIHKNWF | 1.4833 | -0.0542 | 0.08278 | × |
|  | IFNYLFNTL | 1.4381 | 0.1686 | 0.1108 | × |
|  | IFAAIIFAL | 1.4069 | 0.6140 | 0.42861 | √ |
|  | AYLLLPLFY | 1.3431 | 0.2645 | 0.02304 | × |
|  | YWTILIYSI | 1.341 | 0.4129 | 0.16122 | √ |
|  | VYKAQYQKV | 1.2915 | -0.2294 | -0.37067 | × |
|  | LYGSCQDVI | 1.2745 | -0.6093 | -0.27417 | × |
|  | NYKMICQCI | 1.2612 | 0.8418 | -0.29711 | × |
|  | ELHNCIGPY | 2.1769 | -0.2937 | 0.09889 | × |
|  | SLKKNSGPY | 1.7545 | 0.1685 | -0.42691 | × |
|  | NLPHVVPRY | 1.7519 | 0.6878 | 0.12889 | √ |
|  | FLIKNEIEY | 1.6449 | -0.2021 | 0.08497 | × |
|  | ELADDYIEM | 1.6444 | 0.2601 | 0.22396 | × |
|  | NTIPAISGL | 1.6216 | 0.7847 | 0.0756 | √ |
|  | EISKTGSNM | 1.6088 | 0.6078 | -0.3444 | × |
|  | FTIAICTSF | 1.5805 | -0.0935 | 0.09752 | × |
|  | DARNDLIPY | 1.4476 | 0.9901 | 0.12729 | √ |
|  | FTAAILTGL | 1.422 | 0.3683 | 0.22379 | × |
|  | SIVKSTTAF | 1.4176 | 0.2296 | -0.27254 | × |
|  | EIILSSTKL | 1.4146 | 0.9687 | -0.37803 | × |
|  | STEGIINGF | 1.3998 | -0.9115 | 0.33582 | × |
|  | ETVQKGQKL | 1.3518 | 0.8060 | -0.50502 | × |
|  | VPRYHSIDV | 1.5428 | 1.5628 | 0.01413 | √ |
|  | MPYSMIEAM | 1.5409 | 0.3288 | -0.10603 | × |
|  | VPRMYTCRA | 1.4505 | -1.9096 | -0.14222 | × |
|  | KPYNQKIDL | 1.2824 | 1.0595 | -0.19823 | × |
|  | IPAISGLGV | 1.2807 | 1.2054 | 0.02786 | √ |
|  | YLKNHNIDL | 1.4895 | 1.7400 | 0.07418 | √ |
|  | NIKITYQEL | 1.4518 | 1.8701 | 0.05898 | √ |
|  | VLKQFTIVM | 1.3929 | 0.3190 | 0.10042 | × |
|  | RRKDVIVRK | 1.9196 | -0.4935 | 0.18288 | × |
|  | FRNTKILLF | 1.6207 | 0.8368 | -0.0636 | × |
|  | YRPGLKVDL | 1.4668 | 0.2988 | -0.1355 | × |
|  | HRGCIRFFK | 1.3112 | -0.3854 | 0.30227 | × |
|  | YQMVGVLKF | 1.5206 | 0.4997 | -0.07896 | × |
|  | HHYKPTKNL | 2.4692 | 0.2939 | -0.37824 | × |
|  | NHFRNTKIL | 2.2424 | 1.3442 | 0.01608 | √ |
|  | INDVGNTAL | 1.81 | 0.5454 | 0.13127 | √ |
|  | TRLDARNDL | 1.6397 | 0.8640 | 0.11304 | √ |
|  | AQDAHRMIL | 1.5957 | -0.5836 | 0.05635 | × |
|  | YRSCSMCGL | 1.5952 | 1.4553 | -0.46005 | × |
|  | YRSDFYTII | 1.5827 | 0.0200 | 0.18966 | × |
|  | NKDNAEIIL | 1.5325 | 0.3934 | 0.32312 | × |
|  | SRFSCTEAL | 1.4085 | 0.6582 | -0.03707 | × |
|  | YNVAWRTYL | 1.4027 | 0.4500 | 0.34779 | √ |
|  | TTAFSKNTL | 1.3332 | 0.2110 | -0.21638 | × |
|  | CQDVIRPEL | 1.3177 | -0.4104 | 0.2762 | × |
|  | AFSKNTLAL | 1.297 | 0.1430 | -0.22696 | × |
|  | FQNTQTNQL | 1.2956 | 0.0754 | -0.11244 | × |
|  | DEIGECITL | 1.84 | 0.1219 | 0.25905 | × |
|  | FENTIFNYL | 1.8248 | 0.0044 | 0.26914 | × |
|  | KEMNLSVGL | 1.664 | 1.5802 | -0.1754 | × |
|  | IESEIGTDL | 1.6254 | 0.1383 | 0.25427 | × |
|  | KECMSPESL | 1.6035 | 0.7661 | -0.3779 | × |
|  | CEDFISNIV | 1.3477 | -0.5932 | 0.17117 | × |
|  | LEPIIGDKL | 1.3425 | 0.4388 | 0.18454 | √ |
|  | LEDKAWPIV | 1.2886 | 1.4895 | 0.10521 | √ |
|  | CEEYGCLAI | 1.268 | 1.6829 | 0.02453 | √ |
|  | KSVKFHACF | 1.8987 | -0.0077 | -0.05763 | × |
|  | KSYIAYLLL | 1.632 | 0.5414 | 0.1515 | √ |
|  | LALEATTKF | 1.5249 | 0.8259 | 0.07855 | √ |
|  | NSGPYNVAW | 1.4944 | 0.8498 | 0.04785 | √ |
|  | LSSTKLNHF | 1.3925 | 0.8313 | -0.22164 | × |
|  | IAYLLLPLF | 1.3683 | 0.0208 | -0.04944 | × |
|  | FSKEHTSSW | 1.3599 | 0.1615 | -0.13749 | × |
|  | GSNMGQMTF | 1.3462 | 0.9938 | -0.38036 | × |
|  | LGVGYHLGF | 1.2767 | 0.8511 | 0.08479 | √ |
|  | FFKGTYMPY | 1.426 | 1.4932 | -0.15626 | × |
|  | RQHRGCIRF | 1.4217 | 1.0559 | 0.18739 | √ |
|  | NQLPVLMAY | 1.3364 | 0.5982 | -0.11034 | × |
|  | YYKANAAAY | 1.3302 | 0.4780 | 0.05578 | √ |
|  | EQQSSGFEY | 1.3238 | 0.5891 | -0.17597 | × |
|  | MAYKKGKVY | 1.267 | 0.3122 | -0.55418 | × |
|  | RMGKDCLGY | 1.2624 | -0.3021 | -0.22471 | × |
|  | AISGLGVGY | 1.2505 | 1.1548 | 0.05614 | √ |
