# Supplementary material for: Immunoinformatics Approach for Epitope-Based Peptide Vaccine Design and Active Site Prediction against Polyprotein of Emerging Oropouche Virus
Source: J Immunol Res. 2018 Oct 8;2018:6718083. doi: 10.1155/2018/6718083 (PMC6196980; doi:10.1155/2018/6718083)
Supplement: Supplementary 8 — Table S2: 37 CD8+ T-cell epitopes and their combined score, antigenicity, immunogenicity, conservancy, and MHC-I interaction. [file 6718083.f8.docx]

**Table S2:** 37 CD8+ T-cell epitopes and their combined score, antigenicity, immunogenicity, conservancy and MHC-I interaction.

| NO. | Epitopes | Combined score | Antigenicity | Immunogenicity | MHC-I interaction with an affinity of IC50 < 200 and total score | Conservancy | Position |
| --- | --- | --- | --- | --- | --- | --- | --- |
|  | TSSWGCEEY | 2.1681 | 0.9867 | 0.29444 | HLA-B*27:20, 8.37 (1.73)  HLA-C*03:03, 14.05(1.51)  HLA-A*32:07, 14.19(1.50)  HLA-A*68:23, 22.63(1.30)  HLA-C*12:03, 23.03(1.29)  HLA-B*40:13, 65.91(0.84)  HLA-A*32:15, 101.22(0.65)  HLA-C*07:01, 104.75(0.64)  HLA-B*15:02, 126.83(0.55)  HLA-B*15:17, 136.68(0.52)  HLA-B*35:01, 180.13(0.40) | 100.00% | 1043-1051 |
|  | FSIILGIAY | 2.1233 | 1.0143 | 0.3334 | HLA-B*27:20, 6.63(1.86)  HLA-B*35:01,10.01(1.68)  HLA-B*15:17, 13.08(1.56)  HLA-A*68:23, 15.51(1.49)  HLA-C*03:03, 19.53(1.39)  HLA-A*32:15, 25.48(1.27)  HLA-C*12:03, 26.25(1.26)  HLA-A*32:07, 33.97(1.15)  HLA-B*40:13,39.44(1.08)  HLA-B*15:02, 39.92(1.08)  HLA-B*15:01,78.52(0.78)  HLA-B*15:03, 82.05(0.76)  HLA-A*29:02, 83.13(0.76)  HLA-C*14:02, 102.00(0.67)  HLA-A*01:01, 141.43(0.53)  HLA-A*26:02, 156.43 (0.48) | 4.76% | 7-15 |
|  | CSMCGLIHY | 1.9016 | 0.5400 | 0.04253 | HLA-B*27:20, 9.21 (1.79)  HLA-A*68:23, 15.55(1.56)  HLA-C*12:03, 16.68(1.48)  HLA-A*32:07, 18.88(0.97)  HLA-B*15:17, 59.94 (0.83)  HLA-B*40:13, 84.51 (0.73)  HLA-B*15:02, 104.53 (0.70)  HLA-B*15:01, 112.20 (0.70)  HLA-C*14:02, 112.88(0.67)  HLA-A*32:15, 122.25(0.63)  HLA-B*15:03, 131.25(0.45)  HLA-A*30:02, 198.82(-0.76) | 100.00% | 48-56 |
|  | ALEATTKFY | 1.5358 | 0.6372 | 0.03261 | HLA-B*27:20, 16.88(1.50)  HLA-C*05:01, 25.98(1.32)  HLA-C*12:03, 28.46(1.28)  HLA-A*32:07, 33.73(1.20)  HLA-A*68:23, 37.91(1.15)  HLA-C*03:03, 110.06(0.69)  HLA-C*14:02, 123.49(0.64)  HLA-A*32:15, 148.00(0.56) | 61.90% | 68-76 |
|  | LAIDTGCLY | 1.2597 | 0.9551 | 0.08324 | HLA-C*03:03, 8.35(1.79)  HLA-C*12:03, 9.53(1.73)  HLA-B*15:17, 10.93(1.67)  HLA-A*68:23, 17.90(1.46)  HLA-B*35:01, 25.92(1.30)  HLA-A*32:07, 28.58(1.25)  HLA-A*29:02, 73.92(0.84)  HLA-B*15:01, 122.74(0.62)  HLA-B*15:03, 136.49(0.57)  HLA-B*15:02, 160.04(0.50)  HLA-B*40:13, 169.80(0.48) | 100.00% | 4-12 |
|  | LLLPLFYPV | 1.4238 | 0.4221 | 0.07504 | HLA-A*02:12, 0.90(0.95)  HLA-A*02:06, 1.61(0.69)  HLA-A*02:01, 1.84(0.64)  HLA-A*02:16, 2.08(0.58)  HLA-A*02:11, 2.49(0.50)  HLA-A*02:19, 3.85(0.32)  HLA-A*02:02, 9.89(-0.09)  HLA-A*02:50, 12.45(-0.19)  HLA-B*27:20, 14.50(-0.26)  HLA-A*02:03, 17.95(-0.35)  HLA-A*32:07, 26.31(-0.52)  HLA-A*02:17, 31.09(-0.59)  HLA-C*12:03, 55.62(-0.84)  HLA-A*69:01, 73.94(-0.97)  HLA-A*68:23, 90.31(-1.05)  HLA-C*14:02, 91.96(-1.06)  HLA-C*03:03, 106.07(-1.12)  HLA-B*15:03, 172.22(-1.34)  HLA-A*32:15, 189.34(-1.38) | 61.90% | 26-34 |
|  | SLIEICITL | 1.4066 | 0.9867 | 0.3578 | HLA-A*02:11, 4.70(1.40)  HLA-A*32:07, 8.34(1.15)  HLA-A*02:12, 14.41(0.92)  HLA-A*02:16, 15.36(0.89)  HLA-A*02:01, 15.82(0.88)  HLA-A*68:23, 23.97(0.70)  HLA-A*02:02, 25.47(0.67)  HLA-A*02:50, 25.78(0.66)  HLA-B*27:20, 26.82(0.65)  HLA-C*03:03, 39.41(0.48)  HLA-A*02:03, 52.11(0.36)  HLA-A*02:06, 78.44(0.18)  HLA-C*12:03, 83.79(0.15)  HLA-A*02:17, 88.04(0.13)  HLA-B*15:02, 98.45(-0.10)  HLA-C*07:02, 148.31(-0.10)  HLA-C*14:02, 149.84(-0.10)  HLA-A*02:19, 163.21(-0.14)  HLA-A*32:15, 179.99(-0.18) | 76.19% | 34-42 |
|  | IIFSIILGI | 1.3102 | 0.5287 | 0.13685 | HLA-A*02:50,10.82(0.38)  HLA-A*02:11,11.54(0.35)  HLA-B*40:13,12.39(0.32)  HLA-C*12:03,12.86(0.3)  HLA-B*27:20,19.84(0.12)  HLA-A*32:07,23.28(0.05)  HLA-A*02:03,82.59(-0.50)  HLA-A*02:01,94.65(-0.56)  HLA-A*32:15,110.98(-0.63)  HLA-A*02:02,112.98(-0.64)  HLA-B*15:17,119.59(-0.66)  HLA-A*68:23,132.97(-0.71)  HLA-A*02:06,135.07(-0.72)  HLA-A*68:02,177.66(-0.84)  HLA-A*32:01,179.45(-0.84) | 4.76% | 5-13 |
|  | VMLVIILIL | 1.2554 | 0.5008 | 0.36122 | HLA-B*27:20,5.72(1.23)  HLA-A*02:50,24.84(0.59)  HLA-A*32:07,25.83(0.57)  HLA-A*02:11,42.69(0.35)  HLA-A*68:23,44.03(0.34)  HLA-A*02:16,47.15(0.31)  HLA-A*02:01,47.33(0.31)  HLA-A*02:12,62.33(0.19)  HLA-C*14:02,62.9(0.18)  HLA-A*02:19,65.42(0.17)  HLA-B*40:13,72.6(0.12)  HLA-B*15:03,122.21(-0.1)  HLA-C*03:03,140.16(-0.16)  HLA-A*02:02,155.96(-0.21)  HLA-B*15:02,173.87(-0.26) | 4.76% | 21-29 |
|  | IIFALIITK | 1.6291 | 0.7267 | 0.32685 | HLA-C*12:03,12.54(0.23)  HLA-A*11:01,14.63(0.17)  HLA-A*32:07,22.24(-0.02)  HLA-B*40:13,22.44(-0.02)  HLA-A*03:01,27.91(-0.11)  HLA-B*27:20,35.44(-0.22)  HLA-A*68:23,39.51(-0.27)  HLA-A*68:01,41.93(-0.29)  HLA-A*32:15,95.33(-0.65)  HLA-C*03:03,113.92(-0.73)  HLA-A*30:01,173.77(-0.91) | 80.95% | 12-20 |
|  | LIHYRPGLK | 1.4059 | 1.7718 | 0.06886 | HLA-A*32:07,15.6(-0.23)  HLA-B*27:20,20.16(-0.34)  HLA-C*12:03,52.02(-0.75)  HLA-C*03:03,61.89(-0.82)  HLA-A*68:23,68.83(-0.87)  HLA-A*30:01,80.9(-0.94)  HLA-A*03:01,84.49(-0.96)  HLA-B*40:13,97.26(-1.02)  HLA-A*32:15,100.75(-1.04) | 80.95% | 53-61 |
|  | RVNHFRNTK | 1.3414 | 0.4608 | 0.21039 | HLA-A*68:23,7.32(0.42)  HLA-A*32:07,11.97(0.21)  HLA-A*30:01,23.12(-0.08)  HLA-C*12:03,29.05(-0.18)  HLA-A*11:01,29.19(-0.18)  HLA-A*31:01,36.68(-0.28)  HLA-B*27:20,43.6(-0.35)  HLA-C*05:01,69.13(-0.55)  HLA-A*02:17,88.44(-0.66)  HLA-A*03:01,89.5(-0.67)  HLA-C*03:03,187.33(-0.99) | 42.86% | 36-44 |
|  | TLALEATTK | 1.3029 | 1.3929 | 0.19131 | HLA-C*03:03,11.26(0.18)  HLA-B*27:20,11.87(0.16)  HLA-A*32:07,20.85(-0.09)  HLA-A*68:23,35.71(-0.32)  HLA-C*12:03,74.34(-0.64)  HLA-A*32:15,171.89(-1)  HLA-A*03:01,186.98(-1.04)  HLA-B*40:13,190.08(-1.05) | 4.76% | 66-74 |
|  | KFGPRVNHF | 1.6175 | 1.4607 | 0.10254 | HLA-B*27:20,5.36(1.78)  HLA-A*32:07,12.33(1.42)  HLA-A*24:03,15.72(1.31)  HLA-B*40:13,23.17(1.14)  HLA-C*07:02,34.29(0.97)  HLA-A*02:50,37.6(0.93)  HLA-A*32:15,41.81(0.89)  HLA-A*68:23,44.64(0.86)  HLA-C*12:03,75.37(0.63)  HLA-C*14:02,94.32(0.53) | 33.33% | 32-40 |
|  | IFAAIIFAL | 1.4069 | 0.6140 | 0.42861 | HLA-C*03:03,6.22(1.06)  HLA-A*02:50,9.73(0.86)  HLA-B*27:20,21.06(0.53)  HLA-A*02:02,25.7(0.44)  HLA-A*68:23,38.53(0.26)  HLA-B*40:13,40.45(0.24)  HLA-A*32:07,58.76(0.08)  HLA-A*02:11,80.6(-0.06)  HLA-A*02:06,109.53(-0.19)  HLA-B*15:02,114.88(-0.21)  HLA-C*14:02,117.39(-0.22)  HLA-B*42:01,128.16(-0.26)  HLA-C*12:03,150.39(-0.33)  HLA-C*07:02,156.02(-0.34)  HLA-A*32:15,160.42(-0.36)  HLA-A*68:02,165.8(-0.37)  HLA-A*02:17,196.18(-0.44) | 80.95% | 8-16 |
|  | YWTILIYSI | 1.341 | 0.4129 | 0.16122 | HLA-B*27:20,13.17(0.29)  HLA-A*02:50,14.04(0.27)  HLA-A*24:03,21.31(0.09)  HLA-A*32:07,29.31(-0.05)  HLA-A*32:15,39.56(-0.18)  HLA-B*40:13,43.14(-0.22)  HLA-C*12:03,47.12(-0.26)  HLA-A*68:23,56.85(-0.34)  HLA-C*03:03,62.61(-0.38)  HLA-C*14:02,79.18(-0.48)  HLA-A*02:02,149.97(-0.76)  HLA-A*23:01,169.21(-0.81) | 52.38% | 44-52 |
|  | NLPHVVPRY | 1.7519 | 0.6878 | 0.12889 | HLA-B*27:20,10.63(1.72)  HLA-A*32:07,21.23(1.42)  HLA-A*68:23,26.47(1.33)  HLA-C*12:03,28.85(1.29)  HLA-A*02:17,45.57(1.09)  HLA-B*40:13,60.39(0.97)  HLA-C*14:02,70.73(0.9)  HLA-C*07:02,101.66(0.74)  HLA-A*32:15,122.53(0.66)  HLA-B*15:02,196.89(0.46) | 80.95% | 25-33 |
|  | NTIPAISGL | 1.6216 | 0.7847 | 0.0756 | HLA-A*68:23,11.09(0.85)  HLA-C*03:03,13.76(0.75)  HLA-A*68:02,23.75(0.52)  HLA-A*02:50,30.85(0.4)  HLA-A*32:07,33.27(0.37)  HLA-B*15:02,39.19(0.3)  HLA-B*27:20,41.73(0.27)  HLA-A*26:02,45.53(0.23)  HLA-C*12:03,71.65(0.04)  HLA-A*02:17,110.07(-0.15)  HLA-A*02:06,139.49(-0.25)  HLA-A*69:01,139.59(-0.25)  HLA-C*07:02,155.66(-0.3)  HLA-A*32:15,161.16(-0.31)  HLA-B*15:17,182.69(-0.37) | 80.95% | 54-62 |
|  | DARNDLIPY | 1.4476 | 0.9901 | 0.12729 | HLA-A*68:23,8.24(1.38)  HLA-C*12:03,9.78(1.3)  HLA-A*26:02,19.97(0.99)  HLA-B*35:01,45.25(0.64)  HLA-A*32:07,49.66(0.6)  HLA-A*32:15,57.18(0.53)  HLA-B*27:20,100.11(0.29)  HLA-C*14:02,136.97(0.16) | 4.76% | 7-15 |
|  | VPRYHSIDV | 1.5428 | 1.5628 | 0.01413 | HLA-A*68:23,11.01(-0.14)  HLA-B*27:20,18.68(-0.37)  HLA-C*12:03,20.9(-0.42)  HLA-A*32:07,28.32(-0.55)  HLA-B*07:02,45.59(-0.76)  HLA-B*42:01,81.99(-1.01) | 80.95% | 30-38 |
|  | IPAISGLGV | 1.2807 | 1.2054 | 0.02786 | HLA-A*02:50,8.61(0.11)  HLA-B*27:20,12.6(-0.05)  HLA-A*32:07,27.67(-0.4)  HLA-A*68:23,31.24(-0.45)  HLA-C*12:03,33.51(-0.48)  HLA-B*42:01,54.29(-0.69)  HLA-B*07:02,82.2(-0.87)  HLA-B*40:13,96.59(-0.94)  HLA-A*32:15,99.6(-0.95) | 76.19% | 56-64 |
|  | YLKNHNIDL | 1.4895 | 1.7400 | 0.07418 | HLA-C*12:03,25.02(0.29)  HLA-B*15:02,28.59(0.24)  HLA-A*02:50,33.21(0.17)  HLA-A*68:23,34.18(0.16)  HLA-A*32:07,36.23(0.13)  HLA-C*14:02,53.04(-0.03)  HLA-C*03:03,56.31(-0.06)  HLA-B*27:20,80.63(-0.21)  HLA-A*02:12,105.36(-0.33)  HLA-A*02:17,107.31(-0.34)  HLA-A*02:03,108.63(-0.34)  HLA-C*06:02,140.54(-0.46)  HLA-A*32:15,143.96(-0.47)  HLA-B*08:01,150.5(-0.49)  HLA-A*02:02,152.76(-0.49)  HLA-C*07:02,195.51(-0.6) | 71.43% | 26-34 |
|  | NIKITYQEL | 1.4518 | 1.8701 | 0.05898 | HLA-A*02:50,3.43(1.46)  HLA-C*12:03,11.59(0.93)  HLA-B*27:20,13.63(0.86)  HLA-A*02:17,16.28(0.78)  HLA-A*32:07,36.15(0.44)  HLA-B*15:02,39.47(0.4)  HLA-A*68:23,67.1(0.17)  HLA-C*03:03,80.84(0.09)  HLA-B*40:13,86.28(0.06)  HLA-C*07:02,152.46(-0.19) | 9.52% | 489-497 |
|  | NHFRNTKIL | 2.2424 | 1.3442 | 0.01608 | HLA-B*27:20,4.62(1.46)  HLA-B*40:13,14.52(0.96)  HLA-A*02:50,15.75(0.92)  HLA-A*32:07,23.07(0.76)  HLA-A*68:23,27.65(0.68)  HLA-C*12:03,36.16(0.56)  HLA-B*15:02,49.34(0.43)  HLA-B*15:09,87.36(0.18)  HLA-B*39:01,89.21(0.17)  HLA-C*14:02,126.95(0.02)  HLA-C*07:02,135.26(-0.01)  HLA-A*02:17,139.21(-0.02)  HLA-C*03:03,143.75(-0.04)  HLA-B*38:01,171.42(-0.11)  HLA-C*07:01,173.85(-0.12) | 33.33% | 38-46 |
|  | INDVGNTAL | 1.81 | 0.5454 | 0.13127 | HLA-C*12:03,18.42(0.47)  HLA-A*68:23,20.83(0.41)  HLA-B*27:20,23.74(0.36)  HLA-A*02:50,28.39(0.28)  HLA-B*15:02,37.26(0.16)  HLA-A*32:07,40.74(0.12)  HLA-C*14:02,74.75(-0.14)  HLA-B*40:13,82.21(-0.18)  HLA-C*05:01,96.31(-0.25) | 33.33% | 20-28 |
|  | TRLDARNDL | 1.6397 | 0.8640 | 0.11304 | HLA-C*03:03,10.27(0.82)  HLA-B*27:20,22.67(0.48)  HLA-C*07:01,22.92(0.47)  HLA-A*32:07,30.34(0.35)  HLA-C*06:02,31.83(0.33)  HLA-A*68:23,36.2(0.27)  HLA-A*02:50,48.22(0.15)  HLA-C*07:02,56.52(0.08)  HLA-C*14:02,60.76(0.05)  HLA-B*15:02,68.9(-0.01)  HLA-B*40:13,102.79(-0.18)  HLA-C*12:03,144.95(-0.33) | 4.76% | 4-12 |
|  | YNVAWRTYL | 1.4027 | 0.4500 | 0.34779 | HLA-C*03:03,7.72(0.86)  HLA-A*02:50,11.75(0.68)  HLA-B*27:20,21.01(0.42)  HLA-B*15:02,22.14(0.4)  HLA-A*68:23,22.27(0.4)  HLA-A*32:15,49.8(0.05)  HLA-C*12:03,64.6(-0.06)  HLA-C*14:02,69.28(-0.1)  HLA-A*32:07,70.15(-0.1)  HLA-A*02:17,163.18(-0.47) | 71.43% | 19-27 |
|  | LEPIIGDKL | 1.3425 | 0.4388 | 0.18454 | HLA-A*02:17,6.8(1.03)  HLA-A*02:50,8.5(0.94)  HLA-C*12:03,40.48(0.26)  HLA-A*32:07,44.16(0.22)  HLA-A*68:23,55.69(0.12)  HLA-B*40:13,55.71(0.12)  HLA-B*27:20,73.2(0)  HLA-B*40:01,88.9(-0.08)  HLA-A*32:15,122.25(-0.22)  HLA-B*15:02,191.09(-0.41) | 80.95% | 54-62 |
|  | LEDKAWPIV | 1.2886 | 1.4895 | 0.10521 | HLA-C*12:03,5.54(0.38)  HLA-A*02:50,15.35(-0.06)  HLA-A*32:07,32.81(-0.39)  HLA-A*68:23,48.06(-0.56)  HLA-B*27:20,83.46(-0.79)  HLA-B*40:01,103.01(-0.89)  HLA-A*02:17,129.32(-0.98)  HLA-B*40:13,182.798(-1.14) | 4.76% | 838-846 |
|  | CEEYGCLAI | 1.268 | 1.6829 | 0.02453 | HLA-B*27:20,21.6(-0.01)  HLA-C*12:03,35.83(-0.23)  HLA-A*32:07,46.56(-0.35)  HLA-B*40:13,59.28(-0.45)  HLA-B*40:01,72.43(-0.54)  HLA-A*02:50,82.65(-0.6)  HLA-A*68:23,83.13(-0.6)  HLA-C*05:01,87.63(-0.62)  HLA-A*32:15,111.49(-0.73)  HLA-C*03:03,117.65(-0.75) | 95.24% | 1048-1056 |
|  | KSYIAYLLL | 1.632 | 0.5414 | 0.1515 | HLA-B*15:17,0.75(2.13)  HLA-B*27:20,4.44(1.36)  HLA-A*02:50,6.37(1.2)  HLA-A*32:07,16.29(0.79)  HLA-B*40:13,20.79(0.69)  HLA-B*58:01,53.99(0.27)  HLA-C*12:03,60.15(0.22)  HLA-C*15:02,68.46(0.17)  HLA-A*02:17,78.64(0.11)  HLA-B*15:02,81.7(0.09)  HLA-A*68:23,88.46(0.06)  HLA-A*30:01,92.89(0.04)  HLA-A*32:15,135.91(-0.13)  HLA-C*03:03,150.18(-0.17)  HLA-C*14:02,171.25(-0.23)  HLA-A*32:01,199.96(-0.3) | 33.33% | 20-28 |
|  | LALEATTKF | 1.5249 | 0.8259 | 0.07855 | HLA-B*15:03,16.45(1.18)  HLA-A*68:23,21.97(1.05)  HLA-B*15:17,27.33(0.96)  HLA-A*32:07,32.74(0.88)  HLA-B*35:01,41.55(0.77)  HLA-C*03:03,49.05(0.7)  HLA-C*12:03,56(0.65)  HLA-B*27:20,78.98(0.5)  HLA-A*02:50,161.15(0.19)  HLA-B*15:02,181.23(0.14) | 38.10% | 67-75 |
|  | NSGPYNVAW | 1.4944 | 0.8498 | 0.04785 | HLA-B*27:20,6.75(1.11)  HLA-A*32:07,25.01(0.54)  HLA-C*12:03,25.31(0.54)  HLA-B*40:13,34.59(0.4)  HLA-A*32:15,70.35(0.09)  HLA-A*68:23,74.6(0.07)  HLA-C*07:01,78.01(0.05)  HLA-C*03:03,79.54(0.04)  HLA-B*58:01,87.96(0)  HLA-A*02:50,134.96(-0.19)  HLA-B*57:01,163.24(-0.27) | 80.95% | 15-23 |
|  | LGVGYHLGF | 1.2767 | 0.8511 | 0.08479 | HLA-A*32:15,69.86(0.6)  HLA-C*03:03,74.06(0.57)  HLA-C*12:03,76.77(0.55)  HLA-B*27:20,80.07(0.54)  HLA-A*68:23,102.74(0.43)  HLA-B*15:17,106.83(0.41)  HLA-B*15:03,115.9(0.38)  HLA-C*14:02,146.76(0.27)  HLA-A*02:50,151.08(0.26) | 33.33% | 62-70 |
|  | RQHRGCIRF | 1.4217 | 1.0559 | 0.18739 | HLA-B*27:20,1.95(2.34)  HLA-B*15:03,4.05(2.02)  HLA-A*32:07,4.91(1.94)  HLA-B*40:13,13.39(1.5)  HLA-A*68:23,22.43(1.28)  HLA-B*15:01,71.61(0.78)  HLA-C*12:03,149.35(0.46) | 71.43% | 43-51 |
|  | YYKANAAAY | 1.3302 | 0.4780 | 0.05578 | HLA-C*14:02,2.66(2.34)  HLA-C*12:03,5.17(1.94)  HLA-A*68:23,13.83(1.28)  HLA-C*03:03,17.64(0.46)  HLA-B*27:20,27.7(0.27)  HLA-A*32:07,33.5(0.26)  HLA-B*15:02,54.6(0.13)  HLA-A*32:15,66.72(0.11)  HLA-A*29:02,67.73(0)  HLA-A*24:03,134.44(-1.45) | 33.33% | 627-635 |
|  | AISGLGVGY | 1.2505 | 1.1548 | 0.05614 | HLA-A*32:07,13.84(1.66)  HLA-C*12:03,24.62(1.41)  HLA-A*32:15,26.81(1.37)  HLA-C*03:03,39.05(1.21)  HLA-A*68:23,43.93(1.16)  HLA-B*27:20,59.36(1.03)  HLA-B*40:13,86.28(0.87)  HLA-A*30:02,140.43(0.65)  HLA-B*15:17,186.08(0.53) | 76.19% | 58-66 |
