# Supplementary material for: Immunoinformatics Approach for Epitope-Based Peptide Vaccine Design and Active Site Prediction against Polyprotein of Emerging Oropouche Virus
Source: J Immunol Res. 2018 Oct 8;2018:6718083. doi: 10.1155/2018/6718083 (PMC6196980; doi:10.1155/2018/6718083)
Supplement: Supplementary 9 — Table S3: population coverage for the selected 18 epitopes and their HLA alleles. [file 6718083.f9.docx]

**Table S3:** Population coverage for the selected 18 epitopes and their HLA-alleles.

| **population/area** | **Class I** | | |
| --- | --- | --- | --- |
|  | **Coverage^a^** | **Average_hit^b^** | **pc90^c^** |
| American Samoa | 89.02% | 4.26 | 0.91 |
| American Samoa Polynesian | 89.02% | 4.26 | 0.91 |
| Argentina | 82.14% | 1.32 | 0.56 |
| Argentina Amerindian | 82.14% | 1.32 | 0.56 |
| Australia | 88.75% | 5.22 | 0.89 |
| Australia Australian Aborigines | 75.76% | 4.45 | 0.41 |
| Australia Caucasoid | 99.30% | 6.04 | 2.64 |
| Austria | 95.60% | 2.47 | 1.26 |
| Austria Caucasoid | 95.60% | 2.47 | 1.26 |
| Belgium | 90.39% | 1.67 | 1.01 |
| Belgium Caucasoid | 90.39% | 1.67 | 1.01 |
| Brazil | 93.92% | 5.35 | 1.28 |
| Brazil Amerindian | 86.88% | 4.12 | 0.76 |
| Brazil Caucasoid | 89.70% | 2.15 | 0.97 |
| Brazil Mixed | 95.77% | 5.88 | 1.55 |
| Bulgaria | 89.49% | 3.75 | 0.95 |
| Bulgaria Caucasoid | 82.62% | 1.61 | 0.58 |
| Bulgaria Other | 89.89% | 3.7 | 0.99 |
| Burkina Faso | 82.04% | 2.91 | 0.56 |
| Burkina Faso Black | 82.04% | 2.91 | 0.56 |
| Cameroon | 93.88% | 4.41 | 1.31 |
| Cameroon Black | 93.88% | 4.41 | 1.31 |
| Cape Verde | 92.55% | 2.58 | 1.12 |
| Cape Verde Black | 92.55% | 2.58 | 1.12 |
| Central Africa | 92.35% | 4.31 | 1.17 |
| Central African Republic | 21.21% | 0.25 | 0.13 |
| Central African Republic Black | 21.21% | 0.25 | 0.13 |
| Central America | 4.14% | 0.06 | 0.1 |
| Chile | 92.79% | 4.9 | 1.15 |
| Chile Amerindian | 92.39% | 2.71 | 1.07 |
| Chile Mixed | 89.84% | 4.4 | 0.98 |
| China | 93.35% | 6.33 | 1.25 |
| China Oriental | 93.35% | 6.33 | 1.25 |
| Colombia | 10.94% | 0.16 | 0.11 |
| Colombia Black | 7.15% | 0.13 | 0.11 |
| Colombia Mestizo | 15.54% | 0.18 | 0.12 |
| Croatia | 86.82% | 1.87 | 0.76 |
| Croatia Caucasoid | 86.82% | 1.87 | 0.76 |
| Cuba | 88.45% | 2.14 | 0.87 |
| Cuba Caucasoid | 87.50% | 2.04 | 0.8 |
| Cuba Mulatto | 89.95% | 2.3 | 0.99 |
| Czech Republic | 98.32% | 4.87 | 2.02 |
| Czech Republic Caucasoid | 98.32% | 4.87 | 2.02 |
| East Africa | 93.75% | 3.85 | 1.26 |
| East Asia | 90.66% | 6.11 | 1.06 |
| Ecuador | 79.05% | 1.61 | 0.48 |
| Ecuador Amerindian | 79.05% | 1.61 | 0.48 |
| England | 98.66% | 6.12 | 2.18 |
| England Caucasoid | 99.46% | 7.59 | 2.97 |
| England Jew | 47.87% | 1.17 | 0.19 |
| Equatorial Guinea | 9.71% | 1.42 | 1.21 |
| Equatorial Guinea Black | 9.71% | 1.42 | 1.21 |
| Europe | 98.36% | 7.15 | 2.14 |
| Finland | 99.69% | 8.28 | 3.15 |
| Finland Caucasoid | 99.69% | 8.28 | 3.15 |
| France | 98.40% | 6.77 | 2.16 |
| France Caucasoid | 98.40% | 6.77 | 2.16 |
| Georgia | 95.03% | 6.24 | 1.34 |
| Georgia Caucasoid | 97.03% | 7.09 | 1.63 |
| Georgia Kurd | 79.54% | 3.57 | 0.49 |
| Germany | 99.19% | 7.4 | 2.62 |
| Germany Caucasoid | 99.19% | 7.4 | 2.62 |
| Guatemala | 4.14% | 0.06 | 0.1 |
| Guatemala Amerindian | 4.14% | 0.06 | 0.1 |
| Guinea-Bissau | 88.41% | 2.39 | 0.86 |
| Guinea-Bissau Black | 88.41% | 2.39 | 0.86 |
| Hong Kong | 90.27% | 3.84 | 1.01 |
| Hong Kong Oriental | 90.27% | 3.84 | 1.01 |
| India | 90.28% | 4.94 | 1.02 |
| India Asian | 90.28% | 4.94 | 1.02 |
| Indonesia | 75.39% | 3.27 | 0.41 |
| Indonesia Austronesian | 75.39% | 3.27 | 0.41 |
| Iran | 95.00% | 5.32 | 1.31 |
| Iran Persian | 95.00% | 5.32 | 1.31 |
| Ireland Northern | 99.58% | 6.88 | 3.03 |
| Ireland Northern Caucasoid | 99.58% | 6.88 | 3.03 |
| Ireland South | 99.51% | 6.96 | 2.98 |
| Ireland South Caucasoid | 99.51% | 6.96 | 2.98 |
| Israel | 83.65% | 3.39 | 0.61 |
| Israel Arab | 91.34% | 4.56 | 1.06 |
| Israel Jew | 82.27% | 2.94 | 0.56 |
| Italy | 97.27% | 7.25 | 1.8 |
| Italy Caucasoid | 97.27% | 7.25 | 1.8 |
| Ivory Coast | 45.61% | 0.97 | 0.18 |
| Ivory Coast Black | 45.61% | 0.97 | 0.18 |
| Japan | 89.40% | 6.08 | 0.94 |
| Japan Oriental | 89.40% | 6.08 | 0.94 |
| Jordan | 79.13% | 2.72 | 0.48 |
| Jordan Arab | 79.13% | 2.72 | 0.48 |
| Kenya | 94.81% | 3.8 | 1.36 |
| Kenya Black | 94.81% | 3.8 | 1.36 |
| Korea; South | 93.41% | 6.27 | 1.29 |
| Korea; South Oriental | 93.41% | 6.27 | 1.29 |
| Lebanon | 66.71% | 4.44 | 0.3 |
| Lebanon Mixed | 66.71% | 4.44 | 0.3 |
| Macedonia | 35.73% | 0.78 | 0.16 |
| Macedonia Caucasoid | 35.73% | 0.78 | 0.16 |
| Malaysia | 74.04% | 2.04 | 0.39 |
| Malaysia Austronesian | 69.71% | 3.45 | 0.33 |
| Malaysia Oriental | 79.12% | 1.82 | 0.48 |
| Mali | 95.24% | 4.6 | 1.43 |
| Mali Black | 95.24% | 4.6 | 1.43 |
| Mexico | 94.82% | 5.3 | 1.39 |
| Mexico Amerindian | 97.32% | 5.54 | 1.65 |
| Mexico Mestizo | 84.90% | 1.95 | 0.66 |
| Mongolia | 55.86% | 0.86 | 0.23 |
| Mongolia Oriental | 55.86% | 0.86 | 0.23 |
| Morocco | 96.69% | 4.16 | 1.54 |
| Morocco Arab | 95.56% | 3.28 | 1.38 |
| Morocco Caucasoid | 97.41% | 4.81 | 1.7 |
| New Caledonia | 66.26% | 3.09 | 0.3 |
| New Caledonia Melanesian | 66.26% | 3.09 | 0.3 |
| North Africa | 94.74% | 5.03 | 1.34 |
| North America | 96.49% | 5.49 | 1.57 |
| Northeast Asia | 93.62% | 6.46 | 1.28 |
| Oceania | 81.83% | 4.09 | 0.55 |
| Oman | 89.16% | 1.97 | 0.92 |
| Oman Arab | 89.16% | 1.97 | 0.92 |
| Pakistan | 90.53% | 4.85 | 1.02 |
| Pakistan Asian | 91.13% | 5.17 | 1.05 |
| Pakistan Mixed | 89.12% | 4.14 | 0.92 |
| Papua New Guinea | 79.64% | 4.69 | 0.49 |
| Papua New Guinea Melanesian | 79.64% | 4.69 | 0.49 |
| Peru | 90.96% | 1.63 | 1.02 |
| Peru Amerindian | 90.78% | 1.41 | 1.01 |
| Peru Mestizo | 1.99% | 0.22 | 1.12 |
| Philippines | 91.92% | 7.28 | 1.16 |
| Philippines Austronesian | 91.92% | 7.28 | 1.16 |
| Poland | 98.60% | 7.77 | 2.23 |
| Poland Caucasoid | 98.60% | 7.77 | 2.23 |
| Portugal | 92.99% | 4.64 | 1.14 |
| Portugal Caucasoid | 92.99% | 4.64 | 1.14 |
| Romania | 88.43% | 1.77 | 0.86 |
| Romania Caucasoid | 88.43% | 1.77 | 0.86 |
| Russia | 93.62% | 6.11 | 1.28 |
| Russia Caucasoid | 83.86% | 4.88 | 0.62 |
| Russia Mixed | 73.27% | 5.37 | 0.37 |
| Russia Other | 95.67% | 6.54 | 1.48 |
| Russia Siberian | 93.35% | 5.86 | 1.24 |
| Rwanda | 35.26% | 0.48 | 0.15 |
| Rwanda Black | 35.26% | 0.48 | 0.15 |
| Sao Tome and Principe | 87.39% | 2.31 | 0.79 |
| Sao Tome and Principe Black | 87.39% | 2.31 | 0.79 |
| Saudi Arabia | 96.65% | 5.32 | 1.63 |
| Saudi Arabia Arab | 96.65% | 5.32 | 1.63 |
| Scotland | 56.99% | 3.81 | 0.23 |
| Scotland Caucasoid | 56.99% | 3.81 | 0.23 |
| Senegal | 93.09% | 3.77 | 1.21 |
| Senegal Black | 93.09% | 3.77 | 1.21 |
| Serbia | 50.02% | 0.8 | 0.2 |
| Serbia Caucasoid | 50.02% | 0.8 | 0.2 |
| Singapore | 91.43% | 5.2 | 1.11 |
| Singapore Austronesian | 88.49% | 5.06 | 0.87 |
| Singapore Oriental | 94.25% | 5.72 | 1.38 |
| South Africa | 1.53% | 5.81 | -2396.61 |
| South Africa Black | 92.73% | 2.82 | 1.15 |
| South Africa Other | 95.84% | 5.34 | 1.51 |
| South America | 91.49% | 4.7 | 1.1 |
| South Asia | 91.76% | 5.15 | 1.11 |
| Southeast Asia | 91.49% | 5.52 | 1.12 |
| Southwest Asia | 87.96% | 4.07 | 0.83 |
| Spain | 93.62% | 6.05 | 1.24 |
| Spain Caucasoid | 93.62% | 6.05 | 1.24 |
| Sri Lanka | 48.73% | 0.83 | 0.2 |
| Sri Lanka Asian | 48.73% | 0.83 | 0.2 |
| Sudan | 96.64% | 6.51 | 1.58 |
| Sudan Arab | 74.63% | 1.62 | 0.39 |
| Sudan Black | 2.19% | 0.02 | 0.1 |
| Sudan Mixed | 92.71% | 6.62 | 1.2 |
| Sweden | 96.99% | 3 | 1.53 |
| Sweden Caucasoid | 96.99% | 3 | 1.53 |
| Switzerland | 78.00% | 5.34 | 0.45 |
| Switzerland Caucasoid | 78.00% | 5.34 | 0.45 |
| Taiwan | 93.52% | 5.75 | 1.32 |
| Taiwan Oriental | 93.52% | 5.75 | 1.32 |
| Thailand | 94.51% | 5.79 | 1.4 |
| Thailand Oriental | 94.51% | 5.79 | 1.4 |
| Tunisia | 95.44% | 5 | 1.38 |
| Tunisia Arab | 95.44% | 5 | 1.38 |
| Turkey | 72.22% | 3.84 | 0.36 |
| Turkey Caucasoid | 72.22% | 3.84 | 0.36 |
| Uganda | 95.24% | 4.69 | 1.42 |
| Uganda Black | 95.24% | 4.69 | 1.42 |
| United Arab Emirates | 7.46% | 0.26 | 0.22 |
| United Arab Emirates Arab | 7.46% | 0.26 | 0.22 |
| United Kingdom | 85.71% | 4.21 | 0.7 |
| United Kingdom Caucasoid | 85.71% | 4.21 | 0.7 |
| United States | 96.61% | 5.54 | 1.59 |
| United States Amerindian | 90.99% | 4.01 | 1.05 |
| United States Asian | 94.58% | 6.34 | 1.41 |
| United States Black | 96.18% | 4.71 | 1.58 |
| United States Caucasoid | 98.96% | 7.09 | 2.43 |
| United States Hispanic | 95.86% | 5.58 | 1.48 |
| United States Mestizo | 95.64% | 5.77 | 1.48 |
| United States Polynesian | 93.86% | 8.23 | 1.61 |
| Venezuela | 89.74% | 4.09 | 0.98 |
| Venezuela Amerindian | 90.61% | 4.16 | 1.04 |
| Vietnam | 92.10% | 6.84 | 1.19 |
| Vietnam Oriental | 92.10% | 6.84 | 1.19 |
| Wales | 1.00% | 0.01 | 0.1 |
| Wales Caucasoid | 1.00% | 0.01 | 0.1 |
| West Africa | 91.97% | 3.62 | 1.12 |
| West Indies | 88.14% | 2.13 | 0.84 |
| World | 96.44% | 6.28 | 1.6 |
| Zambia | 97.75% | 4.57 | 1.95 |
| Zambia Black | 97.75% | 4.57 | 1.95 |
| Zimbabwe | 96.30% | 3.95 | 1.54 |
| Zimbabwe Black | 96.30% | 3.95 | 1.54 |

^a^ projected population coverage
^b^ average number of epitope hits / HLA combinations recognized by the population
^c^ minimum number of epitope hits / HLA combinations recognized by 90% of the population
